# Causes of non-malarial fever in Laos: a prospective study

**DOI:** 10.1016/S2214-109X(13)70008-1

**Published:** 2013-07

**Authors:** Mayfong Mayxay, Josée Castonguay-Vanier, Vilada Chansamouth, Audrey Dubot-Pérès, Daniel H Paris, Rattanaphone Phetsouvanh, Jarasporn Tangkhabuanbutra, Phouvieng Douangdala, Saythong Inthalath, Phoutthalavanh Souvannasing, Günther Slesak, Narongchai Tongyoo, Anisone Chanthongthip, Phonepasith Panyanouvong, Bountoy Sibounheuang, Koukeo Phommasone, Michael Dohnt, Darouny Phonekeo, Bouasy Hongvanthong, Sinakhone Xayadeth, Pakapak Ketmayoon, Stuart D Blacksell, Catrin E Moore, Scott B Craig, Mary-Anne Burns, Frank von Sonnenburg, Andrew Corwin, Xavier de Lamballerie, Iveth J González, Eva Maria Christophel, Amy Cawthorne, David Bell, Paul N Newton

**Affiliations:** a Lao Oxford Mahosot Hospital Wellcome Trust Research Unit (LOMWRU), Microbiology Laboratory, Mahosot Hospital, Vientiane, Laos; b Faculty of Postgraduate Studies, University of Health Sciences, Vientiane, Laos; c Centre for Tropical Medicine, Nuffield Department of Clinical Medicine, University of Oxford, Oxford, UK; d UMR_D 190 “Emergence des Pathologies Virales”, Aix-Marseille University, IRD French Institute of Research for Development, EHESP French School of Public Health, Marseille, France; e Mahidol–Oxford Research Unit (MORU), Faculty of Tropical Medicine, Mahidol University, Bangkok; f Luang Namtha Provincial Hospital, Luang Namtha, Luang Namtha Province, Laos; g Salavan Provincial Hospital, Salavan, Salavan Province, Laos; h Tropical Hospital Paul-Lechler-Krankenhaus, Tübingen, Germany; i WHO/FAO/OIE Collaborating Centre for Leptospirosis Reference and Research, Queensland, Australia; j School of Biomedical Sciences, Queensland University of Technology, Queensland, Australia; k National Centre for Laboratory and Epidemiology, Vientiane, Laos; l Centre for Malariology, Parasitology, and Entomology, Vientiane, Laos; m WHO, Vientiane, Laos; n Department of Infectious Diseases and Tropical Medicine, University of Munich, Munich, Germany; o Centers for Disease Control and Prevention, US Embassy, Vientiane, Laos; p Foundation for Innovative New Diagnostics (FIND), Geneva, Switzerland; q WHO–Regional Office for the Western Pacific, Manila, Philippines

## Abstract

**Background:**

Because of reductions in the incidence of *Plasmodium falciparum* malaria in Laos, identification of the causes of fever in people without malaria, and discussion of the best empirical treatment options, are urgently needed. We aimed to identify the causes of non-malarial acute fever in patients in rural Laos.

**Methods:**

For this prospective study, we recruited 1938 febrile patients, between May, 2008, and December, 2010, at Luang Namtha provincial hospital in northwest Laos (n=1390), and between September, 2008, and December, 2010, at Salavan provincial hospital in southern Laos (n=548). Eligible participants were aged 5–49 years with fever (≥38°C) lasting 8 days or less and were eligible for malaria testing by national guidelines.

**Findings:**

With conservative definitions of cause, we assigned 799 (41%) patients a diagnosis. With exclusion of influenza, the top five diagnoses when only one aetiological agent per patient was identified were dengue (156 [8%] of 1927 patients), scrub typhus (122 [7%] of 1871), Japanese encephalitis virus (112 [6%] of 1924), leptospirosis (109 [6%] of 1934), and bacteraemia (43 [2%] of 1938). 115 (32%) of 358 patients at Luang Namtha hospital tested influenza PCR-positive between June and December, 2010, of which influenza B was the most frequently detected strain (n=121 [87%]). Disease frequency differed significantly between the two sites: Japanese encephalitis virus infection (p=0·04), typhoid (p=0·006), and leptospirosis (p=0·001) were more common at Luang Namtha, whereas dengue and malaria were more common at Salavan (all p<0·0001). With use of evidence from southeast Asia when possible, we estimated that azithromycin, doxycycline, ceftriaxone, and ofloxacin would have had significant efficacy for 258 (13%), 240 (12%), 154 (8%), and 41 (2%) of patients, respectively.

**Interpretation:**

Our findings suggest that a wide range of treatable or preventable pathogens are implicated in non-malarial febrile illness in Laos. Empirical treatment with doxycycline for patients with undifferentiated fever and negative rapid diagnostic tests for malaria and dengue could be an appropriate strategy for rural health workers in Laos.

**Funding:**

Wellcome Trust, WHO–Western Pacific Region, Foundation for Innovative New Diagnostics, US Centers for Disease Control and Prevention.

## Introduction

Increased use of rapid diagnostic tests for malaria in Laos has shown that many individuals with suspected malaria are not infected with *Plasmodium falciparum* or *Plasmodium vivax*. Although the incidence of falciparum malaria is falling, transmission is heterogeneous between regions, with higher incidence in the south than in the north.^1^ Therefore, a major clinical question paradoxically arises as malaria diagnosis improves: what are the main diagnoses among febrile patients without malaria and how should these individuals be treated?

What treatment patients without malaria receive at all levels of health care in rural Laos is left to health workers to decide. However, few diagnostic facilities or data are available to identify the pathogens responsible, or their antimicrobial susceptibility patterns, to guide these decisions. A wide range of infectious diseases have been described from Laos, including typhoid, scrub typhus (*Orientia tsutsugamushi*), murine typhus (*Rickettsia typhi*), *Neorickettsia sennetsu*, dengue, leptospirosis, Japanese encephalitis virus, and influenza.^2^, ^3^, ^4^, ^5^, ^6^, ^7^, ^8^, ^9^, ^10^, ^11^ However, most of these data are from Vientiane where no malaria transmission takes place.^1^ In malaria-endemic regions of Laos, village health volunteers are trained to undertake rapid diagnostic tests for malaria and to give antimalarial drugs to patients who test positive. Most results are negative, thus information is needed to develop algorithms to manage febrile patients with no malaria. To be effective, such algorithms should take into account heterogeneity in incidence and epidemiology of infectious disease across a country, because to base empirical treatment on a country-wide protocol could reduce effectiveness.^12^ We therefore did a prospective investigation of the causes of acute fever in patients tested for malaria in northern and southern Laos.

## Methods

### Study design and participants

**Figure 1:**
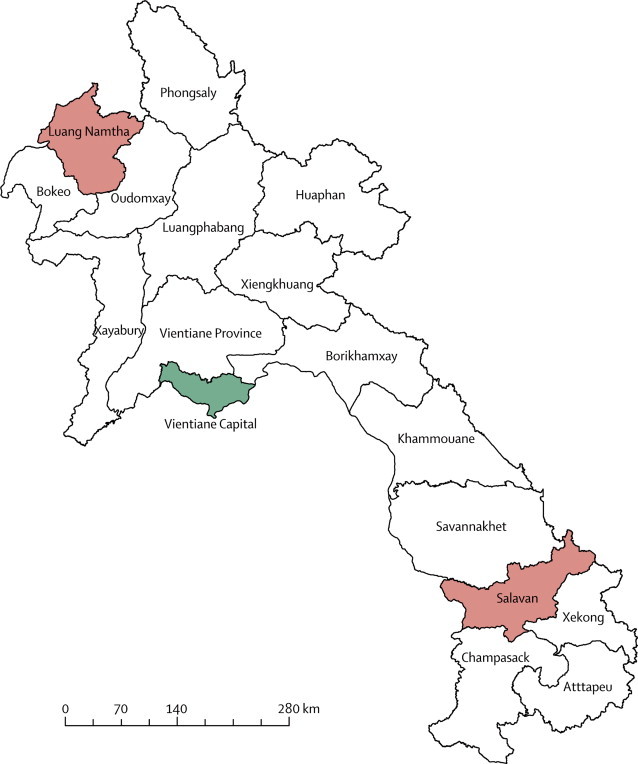
Map of Laos showing location of Vientiane and the study sites in Luang Namtha and Salavan provinces

We did this prospective study at Luang Namtha provincial hospital in northwest Laos between May 2, 2008, and Dec 28, 2010, and at Salavan provincial hospital in southern Laos between Sept 5, 2008, and Dec 26, 2010. The hospitals are roughly 770 km apart. Luang Namtha has 60 beds and serves a population of about 145 000 individuals from the highlands of the China–Burma border. Salavan has 70 beds and serves a population of about 332 000 from the western slopes of the Annamite mountains (figure 1). We recruited inpatients and outpatients aged 5–49 years who gave written informed consent; were eligible for malaria rapid diagnostic testing or microscopy by Lao national guidelines; had no obvious causes of fever (abscess or severe diarrhoea) that would indicate that there was no need for malaria testing; and whose fever had lasted for 8 days or less with an admission tympanic temperature of 38°C or more. We chose, with restricted resources, to recruit patients aged 5–49 years because 75% of Lao patients with malaria are within this age range. Clinicians at both hospitals recorded clinical features of patients at admission and results of routine laboratory investigations in a case record form. Radiological investigations were scarcely available and are not included here. We defined acute encephalitis syndrome as occurring “in a person of any age, at any time of year, with the acute onset of fever and either a change in mental status (including symptoms such as confusion, disorientation, coma, or inability to talk) or new onset of seizures (excluding simple febrile seizures) or both”.^13^ We defined meningitis as a patient with “sudden onset of fever (>38·5°C rectal or 38·0°C axillary) and one of three other signs: neck stiffness, altered consciousness, or other meningeal sign”.^13^

Written informed consent was obtained from all recruited patients or responsible guardians. Ethics approval was obtained from the Lao National Ethics Committee for Health Research and the Oxford Tropical Research Ethics Committee.

### Samples and transport

We requested that all patients provide three blood spots on 903 filter paper (Whatman, Maidstone, UK), and for those aged 5–15 years, 2 mL of whole blood for each of two blood cultures, 3 mL of EDTA blood, and 3 mL of clotted blood. The equivalent volumes for patients older than 15 years were 5 mL of whole blood for each of two blood cultures, 5 mL of EDTA blood, and 5 mL of clotted blood. At 7–14 days' follow-up after discharge, we requested an additional 3 mL of clotted blood for convalescent serology. From May, 2010, nasopharyngeal swabs were taken from all consenting patients at Luang Namtha. Samples were packaged in plastic screw-capped tubes with paper wadding, which in turn were kept in double-skinned locked metal boxes (appendix).

### Laboratory assays

We tested for *Plasmodium* spp, *Leptospira* spp*, O tsutsugamushi, R typhi,* spotted-fever-group rickettsia, causes of community bacteraemia, dengue fever, Japanese encephalitis virus, and, for the last 6 months at Luang Namtha, influenza (appendix). We did not test for tuberculosis or HIV. We followed manufacturer's instructions unless otherwise stated. We did Giemsa-stained malaria smears and plasmodium lactate-dehydrogenase-based immunochromatographic tests (ICT Malaria Combo Cassette Test; ICT Diagnostics, Cape Town, South Africa) for all patients. Full blood counts were done when possible at Salavan (ABX Micros 60 Hematology Analyzer, Horiba ABX, Japan) and Luang Namtha (Mindray BC 3000 Hematology Analyzer, Mindray Medical Instrumentation, NJ, USA). We identified positive blood cultures with conventional techniques^3^ and antibiotic susceptibility by disc diffusion with Clinical and Laboratory Standards Institute criteria.^14^ We did rickettsial culture by inoculation of buffy coat onto Vero and L929 cells with incubation for 6–8 weeks, and speciation by immunofluorescence assay and PCR.^15^ We undertook leptospiral culture with the clot remaining after centrifugation of clotted blood, with Ellinghausen-McCullough-Johnson-Harris medium.^16^ Rickettsial and leptospiral culture began in August, 2009, representing 16 months of the study.

We used dengue and Japanese encephalitis virus ELISAs (Panbio, Brisbane, Australia) to detect dengue NS1, anti-dengue IgM and IgG, and anti-Japanese encephalitis virus IgM (appendix). Immunofluorescence assays were done for antibodies, in dried blood-spot elutes, against *O tsutsugamushi* and *R typhi*. We defined a positive result as an IgM or IgG titre of 1:400 or more.^17^ We regarded leptospiral microscopic agglutination tests as positive if serum showed a titre of 1:400 or more or if paired sera showed a four-fold rise.^9^

We extracted nucleic acids and did all PCRs in duplicate on a Rotor-Gene 3000 or 6000 (Qiagen, Germany) for real-time PCR and a DNA Engine (MJ Research, Canada) for conventional PCR. We based detection of dengue virus on the single-step TaqMan real-time PCR assay.^18^ For *Plasmodium* spp, we used a nested conventional PCR assay^19^ targeting the ssrRNA gene, with distinguishing of *P falciparum* from *P vivax*. For *Leptospira* species, we used a TaqMan real-time PCR assay, detecting the *Leptospira rrs* gene.^20^

We used three probe-based real-time PCR assays to detect *O tsutsugamushi* (47 kDa *htrA* gene), *Rickettsia* genus (17 kDa gene), and *R typhi* (*ompB* gene).^21^, ^22^, ^23^, ^24^ We regarded *Rickettsia* genus 17 kDa real-time PCR-positive samples, and *R typhi ompB* real-time PCR-negative samples as *Rickettsia* spp, which subsequently underwent a panel of nested conventional PCR assays targeting the 17 kDa, *gltA, ompB, ompA,* and *sca4* genes.^22^, ^24^ For positive amplicons, DNA sequencing was done by Macrogen (Seoul, South Korea), followed by Basic Local Alignment Search Tool (BLAST) searches of GenBank. We collected nasopharyngeal or oropharyngeal swabs at Luang Namtha from June, 2010, to December, 2010. The National Centre for Laboratory and Epidemiology did influenza real-time PCR with US Centers for Disease Control and Prevention primers and probes for the influenza virus (H1N1, H3N2, pandemic H1N1 2009, H5N1, and influenza B).^25^

We classified patients' diagnoses in two ways. First, the more conservative, and probably more accurate approach, using only diagnoses based on culture (ie, blood, rickettsial, and leptospiral culture), antigen detection (dengue NS1), and PCR (*Plasmodium* spp, *O tsutsugamushi, R typhi*, spotted-fever-group *Rickettsia* spp, *Leptospira* spp, and dengue) plus, potentially less reliably, anti-Japanese encephalitis virus IgM ELISA.^26^ Second, we used all available tests (ie, the above plus *O tsutsugamushi* and *R typhi* immunofluorescence assay and dengue IgM and IgG ELISAs, which are likely to have lower specificity). Concordance between duplicate PCR assays was high (appendix), except for *R typhi* because of the difficulties in distinguishing *R typhi* from the spotted fever group. We analysed the association between patient symptoms, signs, and laboratory features for each aetiological diagnosis (see statistical analysis).

### Antibiotic use and effectiveness

At Luang Namtha, we recorded patients' use of antibiotics before admission. We derived a rough estimation of the therapeutic effectiveness by consensus among the authors, with use of evidence from Laos as much as possible (appendix p 55). For many pathogens the evidence base is inadequate to provide robust evidence, with confidence intervals, of efficacy or effectiveness.

### Statistical analysis

We analysed data with SPSS and Stata (both version 10). We compared normally distributed data with Student's *t* test, and non-normally distributed data with the Mann-Whitney U test. We compared categorical data with χ^2^ and Fisher's exact tests. To predict factors associated with cause, we included all significant variables from the univariable analysis in a multivariable logistic regression model using a backward stepwise approach; we retained significant variables in the final models. We checked the fit of models with the Hosmer-Lemeshow or Pearson's goodness-of-fit tests.

### Role of the funding source

The sponsors of the study had no role in study design, data collection, data analysis, data interpretation, or writing of the report. The corresponding author had full access to all the data in the study and had final responsibility for the decision to submit for publication.

## Results

We enrolled 1938 patients (1390 at Luang Namtha over 32 months [roughly 43 per month] and 548 patients at Salavan over 28 months [roughly 20 per month]). The median distances from patients' homes to the hospitals were 6 km (range <1–174) for Luang Namtha and 13 km (<1–238) for Salavan (appendix). The overall median age of participants was 19 years (5–49) years; 37% were children aged 15 years or younger and 42% were women (appendix pp 18–22). 1393 (72%) of 1938 patients were seen at follow-up, with a median interval between admission and convalescent samples of 7 days (1–374).

**Figure 2:**
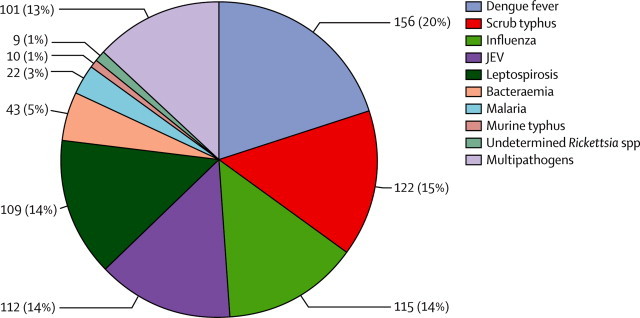
Diagnoses for patients (N=799) at both study sites, with use of only culture, antigen, and nucleic acid detection assays (conservative definition) See table 1 for more information. Influenza diagnosis was only done on samples collected from Luang Namtha for 6 months. JEV=Japanese encephalitis virus.

With conservative definitions of cause, 41% of patients were assigned a diagnosis (table 1). With exclusion of influenza, the top five diagnoses when only one diagnosis per patient was made, were dengue (8%), scrub typhus (7%), Japanese encephalitis virus (6%), leptospirosis (6%), and bacteraemia (2%; table 1, figure 2, appendix). Protozoa (malaria) were responsible for 22 (1%) of 1936 fevers, bacteria for 293 (15%) of 1938, and viruses (excluding influenza) for 268 (14%) of 1938 fevers.

**Table 1:** Overall conservative diagnoses of enrolled patients, with use of only culture, antigen, and nucleic acid assays, plus IgM against Japanese encephalitis virus for patients in whom only one pathogen was detected

		**All (N=1938)**	**Luang Namtha (n=1390)**	**Salavan (n=548)**	**p value**
With diagnosis		799/1938 (41%)	552/1390 (40%)	247/548 (45%)	0·03
Single pathogens					
	Any single pathogen	698/1938 (36%)	473/1390 (34%)	225/548 (41%)	0·004
	Dengue (PCR or dengue virus-NS1, or both)	156/1927 (8%)	41/1382 (3%)	115/545 (21%)	<0·0001
	Scrub typhus (PCR or culture, or both)	122/1871 (7%)	86/1337 (6%)	36/534 (7%)	0·8
	Influenza (PCR)	115/358 (32%)	115/358 (32%)	..	..
	Japanese encephalitis virus (ELISA)	112/1924 (6%)	90/1383 (7%)	22/541 (4%)	0·04
	Leptospirosis (PCR, culture, or MAT*)	109/1934 (6%)	93/1389 (7%)	16/545 (3%)	0·001
	Bacteraemia (culture)	43/1938 (2%)	30/1390 (2%)	13/548 (2%)	0·77
	Malaria (RDT, or smear or PCR, or both)	22/1936 (1%)	4/1308 (<1%)	18/528 (3%)	<0·0001
	Murine typhus (PCR)	10/1849 (<1%)	6/1320 (<1%)	4/529 (<1%)	0·49
	Undetermined *Rickettsia* spp and *Rickettsia felis* (PCR)	9/1849 (<1%)	8/1320 (<1%)	1/529 (<1%)	0·46
Evidence for several pathogens (mixed infections†)					
	More than one pathogen	101/1938 (5%)	79/1390 (6%)	22/548 (4%)	0·14
	Two pathogens	98/1938 (5%)	77/1390 (6%)	21/548 (4%)	0·12
	Three pathogens	3/1938 (<1%)	2/1390 (<1%)	1/548 (<1%)	1·0

Data are n/N (%). Differences in data between this table and the text arise when patients with more than one apparent infection are included. Influenza diagnosis was only done for samples collected from Luang Namtha for 6 months. Comparisons are for unadjusted data.·MAT=microscopic agglutination test. RDT=rapid diagnostic test.

* With a four-fold rise in titre.

† See appendix for more information.

The combination of microscopy, rapid diagnostic tests, and PCR identified only 25 (1%) patients with malaria, (18 falciparum, six vivax, and one mixed; appendix). Clinically significant organisms were grown from the blood cultures of 53 (3%) patients (table 2). The volume of blood added to the blood culture bottles was about 80% of that advised for 2 mL bottles, and 62% of that for 5 mL bottles (appendix). High blood volume was not associated with either the first or second blood culture bottle per patient, or the growth of clinically significant causes of community-acquired bacteraemia (appendix). The most commonly identified pathogen was *Salmonella enterica* serovar Typhi, followed by *Escherichia coli, Burkholderia pseudomallei, Staphylococcus aureus*, and *Klebsiella pneumoniae* (table 2). One isolate of *S typhi* was resistant to nalidixic acid, implying that it would respond poorly to oral fluoroquinolones (appendix).^3^

**Table 2:** Blood culture results

		**All (N=1938)**	**Luang Namtha (n=1390)**	**Salavan (n=548)**	**CRP >5 mg/L (960/1170)**
No growth		1777/1938 (92%)	1302/1390 (94%)	475/548 (87%)	866/960 (90%)
Positive growth		161/1938 (8%)	88/1390 (6%)	73/548 (13%)	94/960 (10%)
Contaminants		106/161 (66%)	51/88 (58%)	55/73 (75%)	..
Uncertain clinical significance (*Leuconostoc* sp and *Achromobacter xylosoxidans*)*		2/161 (1%)	1/88 (1%)	1/73 (1%)	..
Clinically significant organisms		53/161 (33%)	36/88 (41%)	17/73 (23%)	38/40 (95%)
	*Salmonella enterica* Typhi	38/53 (72%)	30/36 (83%)	8/17 (47%)	··
	*Escherichia coli*	4/53 (8%)	2/36 (6%)	2/17 (12%)	··
	*Burkholderia pseudomallei*	3/53 (6%)	0/36	3/17 (18%)	··
	*Klebsiella pneumoniae*	2/53 (4%)	1/36 (3%)	1/17 (6%)	··
	*Staphyloccocus aureus*	2/53 (4%)	1/36 (3%)	1/17 (6%)	··
	*Streptococcus suis*	1/53 (2%)	0/36	1/17 (6%)	··
	*Streptococcus* Group A	1/53 (2%)	0/36	1/17 (6%)	··
	*Streptococcus* Group C	1/53 (2%)	1/36 (3%)	0/17	··
	*Salmonella enterica* Group C	1/53 (2%)	1/36 (3%)	0/17	··

Data are n/N (%), unless otherwise indicated.

* Two organisms, *Leuconostoc* sp^27^ and *Achromobacter xylosoxidans*^28^ were probable contaminants, but because they are very rare causes of bacteraemia, we have classified them as of uncertain clinical significance

Filter paper blood-spot immunofluorescence assay for rickettsial infections showed IgM titres of more than 1:400 against *O tsutsugamushi* in 21% of patients' sera (appendix). That scrub typhus occurred at both study sites was proven by *O tsutsugamushi* culture from 3% of patients (appendix), despite the more than 24 h interval between drawing of blood and culture. No *R typhi* or spotted-fever-group *Rickettsia* species were grown, although *R typhi* has been grown from patients admitted at Mahosot Hospital (Vientiane, Laos). PCR results are in line with those from the immunofluorescence assay. We detected *O tsutsugamushi* in 9% of patients, but *R typhi* in less than 1% (appendix). One additional patient was PCR positive for both *O tsutsugamushi* and *R typhi* (appendix).^26^ *Rickettsia felis* DNA was detected in two patients (both samples were 17 kDa real-time and 17 kDa-nested PCR-positive; confirmatory DNA sequencing for 17 kDa and *sca4* genes showed 100% similarity to *R felis*). Microscopic agglutination tests, culture, and PCR for *Leptospira* species were positive for 7%, 3%, and 4% of patients, respectively (appendix).

Serological evidence for dengue was present for 21% of 1904 patients, and PCR evidence for 8% (appendix). For the 225 patients for whom primary and secondary dengue could be distinguished, 69% were diagnosed with primary dengue and 31% with the secondary form (appendix). IgM against Japanese encephalitis virus was detected in acute serum samples from 8% of 1875 patients, and in convalescent serum samples from 8% of 1357 patients (appendix). Between June and December, 2010, we did influenza PCR of throat swabs from 358 (26%) patients from Luang Namtha, of whom 139 (39%) tested positive. Influenza B was the most frequently detected strain (121 [87%]), followed by influenza A subtype H3 (ten [7%]), and pandemic influenza A H1N1 (eight [6%]; appendix). 137 (99%) of 139 patients who tested positive had data to establish whether they had influenza-like illness as defined by WHO.^29^ Of these patients, 73 (53%) of those with any influenza, 60 (50%) with influenza B, five (50%) with influenza A subtype H3, and eight (100%) with pandemic H1N1 fulfilled criteria for influenza-like illness.

Data were available to define acute encephalitis syndrome for 1157 (60%) patients and meningitis for 1134 (59%) patients, of whom 5% and 6%, respectively, had meningitis and acute encephalitis syndrome (appendix). IgM against Japanese encephalitis virus was detected in sera of 37% of patients with meningitis and 43% of those with acute encephalitis syndrome, with no diagnosis in 43% and 38%, respectively. Representation of scrub typhus, leptospirosis, dengue, and malaria ranged from less than 1% to 7% (appendix). With conservative definitions 101 (5%) of 1938 patients had evidence of several pathogens, mostly (98 [97%] of 101) dual infections. The most frequent combinations were of Japanese encephalitis virus and scrub typhus (n=26), Japanese encephalitis and influenza (n=16), and dengue and scrub typhus (n=10; appendix). With all diagnostic evidence,^26^ 374 (19%) of 1938 patients had evidence of two or more pathogens (appendix).

Leptospirosis (p=0·001), *S typhi* (p=0·006) and Japanese encephalitis virus (p=0·04) were more common at Luang Namtha hospital than at Salavan, whereas dengue and malaria (both p<0·0001) were more common at Salavan (table 1, appendix). At Luang Namtha hospital, 72% of patients were not admitted, and at Salavan 15% were not admitted (p<0·0001; appendix). The causes of fever in children and adults were similar, except at Luang Namtha hospital where leptospirosis was more common and dengue less common in children than in adults (appendix).

**Figure 3:**
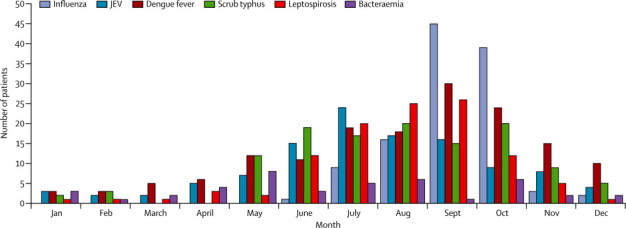
Monthly incidence of diseases, with conservative definitions, for patients from Luang Namtha and Salavan combined Influenza diagnosis was only done on samples collected from Luang Namtha for 6 months. JEV=Japanese encephalitis virus.

Few clinical features accurately predicted the cause of non-malarial fever. Platelets counts of 100 000 per μL or less were associated with dengue (p=0·01). Acute encephalitis syndrome was associated with Japanese encephalitis virus (table 3). Female sex and hepatomegaly were associated with scrub typhus, and an age of 15 years or younger, vomiting, cough, and a C-reactive protein (CRP) concentration of more than 5 mg/L were associated with leptospirosis (table 3). Influenza was associated with retro-orbital pain and cough (table 3; appendix). Scrub typhus, dengue, Japanese encephalitis virus, and leptospirosis all showed seasonality, with much higher hospital presentations in the wettest and hottest season (May to September) than in the cooler months (figure 3, appendix). Community-acquired bacteraemia did not show obvious seasonality (data not shown). Only 6 months of data were obtained for influenza but, during this period, frequency of infection was highest in September to October. Six inpatients died in hospital (appendix). The appendix assesses data for peripheral white blood cell counts and CRP.

**Table 3:** Multivariable logistic regression analysis of predictors significantly associated with detection of pathogens for patients with only evidence for one pathogen with conservative diagnoses

	**Subcategory**	**OR (95% CI)**	**p value**
**Influenza**			
Retro-orbital pain	Yes	2·1 (1·1–3·9)	0·018
Cough	Yes	4·1 (2·6–6·7)	<0·0001
**Japanese encephalitis virus**			
Acute encephalitis syndrome	Yes	5·6 (1·8–17·5)	0·003
Glasgow coma scale	<15/15	4 (1·1–13·9)	0·03
**Dengue**			
Province	Salavan	9·9 (4·8–20·2)	<0·0001
Platelets	≤100 000/μL	4·0 (1·4–11·5)	0·01
**Bacteraemia**			
Arthralgia	Yes	0·4 (0·2–0·9)	0·03
Abdominal pain	Yes	2·4 (1·1–5·2)	0·03
Hepatomegaly	Yes	3·6 (1·2–10·4)	0·02
**Leptospirosis**			
Province	Luang Namtha	1·9 (1·0–3·5)	0·04
Age (years)	≤15	2·2 (1·3–3·6)	0·002
Vomiting	Yes	2·2 (1·3–3·6)	0·003
Cough	Yes	0·2 (0·1–0·5)	<0·0001
C-reactive protein	>5 mg/L	5·3 (1·6–17·2)	0·005
**Scrub typhus**			
Sex	Female	1·8 (1·1–2·8)	0·01
Hepatomegaly	Yes	3·4 (1·6–7·2)	0·001

See appendix for more information. Influenza diagnosis was only done for samples collected from Luang Namtha for 6 months.

Of 1095 (79%) patients at Luang Namtha with data for hospital antibiotic use, 560 (51%) received antibiotics, of whom 67 (12%) received more than one (appendix). Amoxicillin was most commonly used, in 294 (53%) of 560 patients, with doxycycline in 116 (21%). The proportion of patients at Luang Namtha with specific diagnoses who received appropriate antibiotics varied greatly (appendix). Although 51% of patients received an antibiotic, only for 7% was the treatment regarded as appropriate with the benefit of hindsight of the subsequent diagnostic tests. The proportion of patients who received an appropriate treatment varied between 18% for those with a final diagnoses of typhus and 52% for those with leptospirosis (appendix). We used a rough estimation of the therapeutic effectiveness of different antibiotics to estimate the potential public health effect of these regimens in Luang Namtha and Salavan (table 4, appendix). Azithromycin, doxycycline, ceftriaxone, and ofloxacin were estimated to have significant effectiveness for 13%, 12%, 8%, and 2% of patients, respectively (table 4).

**Table 4:** Number of patients expected to respond to empirical treatment

	**Number with potentially antibiotic-susceptible pathogens**	**Oral doxycycline**	**Oral ofloxacin**	**Oral azithromycin**	**Parenteral ceftriaxone**
All (N=1938)	293 (15%)	240 (12%)	41 (2%)	258 (13%)	154 (8%)
Luang Namtha (n=1390)	223 (16%)	184 (13%)	29 (2%)	198 (14%)	125 (9%)
Salavan (n=548)	70 (13%)	56 (10%)	12 (2%)	60 (11%)	29 (5%)

Data are n (%), unless otherwise indicated. Data are for patients with only one pathogen detected. On the basis of assumption of the responses provided in the appendix. The few patients without assays for particular pathogens in appendix p 55 have been excluded. Calculated as the number of patients with each diagnosis multiplied by percentage expected response.

## Discussion

Our findings strongly suggest that leptospirosis and scrub typhus are important and treatable causes of fever in rural Laos and should be considered in any acutely febrile patient with a negative malaria test. The frequency of anti-*R typhi* IgM and IgG was lower than that for *O tsutsugamushi,* which is consistent with evidence that murine typhus is a more urban disease than scrub typhus.^6^ Because mild scrub typhus responds rapidly to oral doxycycline treatment in Vientiane, these data suggest that accessible and inexpensive doxycycline (about Lao Kip [LAK]–10 000, roughly US$1·2, for a 7-day course of 14 tablets) might be an appropriate intervention. Leptospirosis was also an important cause of non-malarial fever in Laos, especially in the north. We would expect mild disease to respond well to oral doxycycline.^30^ Unlike scrub typhus, we would also expect mild forms to respond to oral and parenteral penicillins and cephalosporins. Acute encephalitis syndrome and meningitis are important considerations because empirical treatment for undifferentiated fever might not be effective for these severe syndromes.

Dengue is clearly an important cause of fever outside the few major Lao urban centres, and vector control and training in management of fluid balance are likely to be key interventions. New rapid diagnostic tests for the detection of dengue NS1 have high specificity and sensitivity^31^ and are likely to be helpful for diagnosis of dengue at local hospitals. Japanese encephalitis virus was also an important cause of fever, and vaccines are available with an effectiveness of more than 80% after just one dose.^32^ With an assumption of this level of effectiveness, a vaccine for Japanese encephalitis virus could reduce the frequency of patients developing undifferentiated fever by about 5%. These data, and those describing Japanese encephalitis virus as an important cause of encephalitis in Laos,^10^ suggest that vaccination is likely to reduce the incidence of not only encephalitis, death, and disability, but also undifferentiated fever and the resultant key economic issues of health-care expenditure and loss of work. Vaccination is due to start in Laos in 2013.

With the advent of affordable and accurate rapid diagnostic tests for dengue,^31^ on the basis of detection of dengue NS1 and anti-dengue IgM and IgG antibodies, empirical antibiotic treatment for those with negative dengue and malaria results might be a cost-effective option (panel). Furthermore, with the exception of typhoid, the main treatable fevers were not caused by pathogens diagnosed in conventional microbiology laboratories, but could be diagnosed by combinations of antigen rapid diagnostic tests and simple molecular assays, such as loop-mediated isothermal amplification assays.^33^ Hence, intensive widespread investment in expensive conventional facilities for microbiological culture facilities might not be appropriate.

Panel **Research in context**

**Systematic review**

As described in reference 2, we searched PubMed in English from Jan 1, 1986 to June 9, 2011, with the keywords “Cambodia” or “Lao PDR” (and “Laos”) or “Viet Nam” (and “Vietnam”) or “Myanmar” (and “Burma”) or “Thailand” or “Yunnan Province” (of the People's Republic of China), coupled separately with individual search terms for diseases: “rickettsial infections” (search terms: “rickett*”, “scrub typhus”, “murine typhus”, “spotted fever group rickett*”), “leptospirosis”, “typhoid fever”, “dengue”, “melioidosis”, and “Japanese encephalitis”. For Laos we also searched the grey literature in medical libraries in Vientiane and discussed with local physicians. No studies exist that examine the diversity of bacterial and viral pathogens in one population. Through the above search we identified the potential causes of fever in Laos and the diagnostic and treatment options. Our study adds substantially to the fragmented data previously available and gives the first objective evidence of the causes of fever, when malaria tests are negative, in communities in mainland Asia.

**Interpretation**

Our findings show that a wide range of treatable or preventable pathogens are the cause of fever in patients in rural Laos who present with malaria-like syndromes, but do not have malaria. With exclusion of influenza, the top five diagnoses, when only one aetiological diagnosis per patient was made, were dengue, scrub typhus, Japanese encephalitis virus, leptospirosis, and bacteraemia. Forthcoming Japanese encephalitis vaccination in Laos is likely to reduce the incidence of undifferentiated fever in addition to encephalitis. Significant differences in disease frequency between the two sites, for dengue, typhoid, Japanese encephalitis virus, malaria, and leptospirosis, have important implications for empirical therapy and emphasise the importance of heterogeneity in disease epidemiology within countries. The study suggests that empirical treatment with doxycycline or azithromycin in patients with undifferentiated fever, without malaria, might be a clinically appropriate strategy for reducing morbidity and mortality in rural Laos and elsewhere in mainland southeast Asia. Further cost-effectiveness analysis, including use of rapid diagnostic tests for dengue, would be important to inform policy. More discussion is needed about strategies to build clinically useful and cost-effective country-appropriate laboratory diagnostics to inform treatment and surveillance in rural southeast Asia.

Although described in fleas in Laos^34^ and in one patient at Mahosot Hospital who seroconverted,^5^ our study describes detection of *Rickettsia felis* with molecular techniques for the first time in patients from Laos. *R felis* has been described at the Thailand and Burma border, but not as far as we are aware in Burma, Vietnam, China, or Cambodia.^35^

Other potential uninvestigated infectious causes of undifferentiated fever in Laos include Epstein-Barr virus; hepatitis A, B, C, and E viruses; mycoplasma; cytomegalovirus; hantaviruses; enterovirus; chikungunya; *Coxiella burnetii*; *Brucella* spp; *Neorickettsia sennetsu; Bartonella*; filaria; and diverse respiratory viruses.^36^, ^37^, ^38^, ^39^, ^40^, ^41^, ^42^ As suggested in Cambodia, 50% of cases of influenza B would not have been identified by surveillance for influenza-like illness.^29^ At several Cambodian clinics, causes identified for 38% of febrile patients (fever <10 days, aged >2 years) were mainly influenza, dengue, malaria, and typhoid.^41^ In Papua, Indonesia, the main causes of fever (any age, negative malaria slide) were leptospirosis, rickettsioses, typhoid, and dengue.^42^

Our study has important limitations. First, we did not include diagnosis of urinary tract infections, hepatitis viruses, HIV, and tuberculosis because such diagnosis was only possible in a few patients. At least in Luang Namtha, HIV prevalence is very low.^43^ Second, we did not examine cerebrospinal fluid because such investigations are not possible outside of Vientiane. Third, we did not include patients younger than 5 years or older than 49 years, so generalisability is somewhat restricted. Fourth, in the difficult logistical environment of rural Laos, follow-up was not possible for all patients. Fifth, IgM and IgG anti-dengue antibodies could represent previous infections and not the cause of the presenting illness. Three patients with anti-Japanese encephalitis virus IgM were also dengue PCR-positive, which is suggestive of either mixed infections, ELISA cross-reactivity, or sequential Japanese encephalitis virus and dengue infections. Because we did only 6 months of influenza diagnosis for Luang Namtha, the true importance of influenza will be underestimated. Sixth, the non-incubated transport of blood culture bottles for 24 h before venting and incubation will reduce growth of fastidious organisms such as *Haemophilus influenzae*; however, *S typhi* seemed to tolerate these conditions. Indeed, a greater diversity of pathogens is apparent in blood cultures in Vientiane, presumably because of rapid incubation.^3^ Seventh, antibiotic use before presentation can underestimate the frequency of cultured pathogens from patients with community-acquired bacteraemia.^44^ Eighth, at both hospitals diagnosis of malaria took place during the planning stage of the study, but incidence fell. Ninth, because only a minority of febrile patients would be expected to attend a provincial hospital, selection bias is an important consideration in interpretation of these results. However, in 2009, only 100 (3%) of 2991 patients in all of Luang Namtha, and 2457 (7%) of 33 785 in Salavan were diagnosed with falciparum malaria by microscopy or rapid diagnostic tests (unpublished). More patients were admitted at Salavan than at Luang Namtha, which probably indicates different admission policies. Finally, the disease-specific mortality will be underestimated, with no follow-up data for a substantial number, and because patients are usually taken home to die rather than dying in hospital.

Detection of one or more pathogens is fraught with difficulties if diagnostic techniques that rely on antibody detection or have interspecies cross-reactivity are used. The presence of several pathogens is much more certain if they are grown in culture or shown by specific antigen or nucleic acid detection, or by seroconversion.^26^ The sparse data available for the longevity of specific serum IgM for diseases such as scrub typhus, murine typhus, leptospirosis, and Japanese encephalitis suggest that these immunoglobulins might be detectable for months or perhaps years.^45^, ^46^ Hence, assays based on detection of these IgM antibodies are at risk of being interpreted as mixed infection when they represent previous symptomatic or asymptomatic infection. However, mixed infections of common pathogens, especially for individuals with similar household and occupational exposure, are likely to be frequent.

A further neglected issue in national treatment guidelines is heterogeneity of causes of fever across a country due to environmental and human factors.^12^ Despite being a small country, differences in the cause of disease between north and south Laos are marked. Dengue and malaria were significantly more frequent at Salavan than at Luang Namtha, and leptospirosis, Japanese encephalitis virus, and typhoid were significantly more frequent at Luang Namtha. In view of the very high soil densities of *B pseudomallei* in Salavan,^47^ the incidence of this pathogen is likely to be underestimated in the south. How the effect of policies for treatment of empirical fever will be affected by such heterogeneity needs further investigation.

The relatively high estimated effectiveness of doxycycline results from the high incidence of typhus and leptospirosis, whereas the low estimated effectiveness of ofloxacin is a consequence of its probably low or zero efficacy for typhus and leptospirosis and the low incidence of bacteraemia. The intermediate effectiveness of ceftriaxone results from its efficacy against leptospirosis, which was quite common. The relatively low estimated effects need to be assessed in the context of an extensive diagnostic investigation only giving an aetiological diagnosis for 41% of patients. Doubt remains about the effect of azithromycin for murine typhus and community-acquired bacteraemia, and concerns exist about engenderment of azithromycin resistance. However, if 3 days of azithromycin were effective for these pathogens, this drug might be more effective than doxycycline. Because village health volunteers have only about 2 weeks' training and rural Laos faces economic difficulties, empirical treatment algorithms need to be simple and inexpensive. Addition of another antibiotic, such as ofloxacin, in regions with an increased incidence of bacteraemia such as typhoid, needs detailed cost-effectiveness analysis. The estimated effectiveness of these antibiotics in Salavan was lower than that in Luang Namtha, because of the higher incidence of dengue in Salavan.

Differentiation of patients with different diagnoses, either at the bedside or when all data were analysed, was difficult. A CRP rapid diagnostic test, with a cutoff of 5 mg/L, might identify patients with a bacterial illness and those likely to respond to doxycycline (appendix). Although many patients unlikely to respond to doxycycline would be treated, without expected therapeutic benefit but with risk of adverse effects, few patients likely to respond to doxycycline would be missed by such a strategy. With an assumption that the consequences of no treatment of patients are severe, but the risks and expense of unnecessary antibiotic treatment are moderately low, a diagnostic cutoff to give higher sensitivity at the expense of reduced specificity might be acceptable.

The low proportions of patients who received appropriate treatments for laboratory confirmed typhus and leptospirosis would be increased by empirical doxycycline treatment for undifferentiated fever, especially for those with a negative dengue rapid diagnosis test. Cost-effectiveness analysis of pathogen-specific diagnostics, of CRP, and of other potential severity markers such as procalcitonin is required to inform strategies. Assessment of tests and strategies for both population-wide estimates of incidence and prevalence to inform clinical algorithms, and patient-specific information at the point of care are needed. In the long term, the strengthening of provincial-level, and then district-level, hospitals with an affordable, Lao-appropriate mix of methods for accurate diagnosis of treatable or preventable infectious diseases will hopefully improve understanding of the geographical diversity of pathogens and their optimum treatment.^48^
