# Supplementary material for: Causes of non-malarial fever in Laos: a prospective study
Source: Lancet Glob Health. 2013 Jul;1(3):e46–54. doi: 10.1016/S2214-109X(13)70008-1 (PMC3986032; doi:10.1016/S2214-109X(13)70008-1)

THE LANCET Global Health

Supplementary appendix

This appendix formed part of the original submission and has been peer reviewed. We post it as supplied by the authors.

Supplement to: Mayxay M, Castonguay-Vanier J, Chansamouth V, et al. Causes of non-malarial fever in Laos: a prospective study. *Lancet Glob Health* 2013; **1:** e46–55.

Supplementary Material - Causes of non-malarial fever in Laos: a prospective study

Supplementary Material – 1

Methods

Summary Table

Pathogens	Microscopy	Culture	PCR	IFA/ELISA	Reference
Malaria	Giemsa	-----	Nested conventional PCR for *Plasmodium* ss rRNA on whole blood	----------	Snounou & Singh (2002)[19]
Leptospira	------------	Culture of clot from non-anti-coagulated whole blood clot	TaqMan real-time PCR for *Leptospira* spp. rrs gene on whole blood	----------	Thaipadungpanit *et al.* (2011)[20]<br><br>Wuthiekanun et al. (2007)[16]
Agents of 'conventional' bacteremia	------------	Blood culture with conventional processing for identity and susceptibility pattern	--------------------	------------	Phetsouvanh *et al.* (2006)[3]

R. typhi	------------	Culture of EDTA anti-coagulated buffy coat	TaqMan real-time 17kDa PCR on buffy coat for *Rickettsia* genus and, if positive, TaqMan real-time *ompB* PCR for *R. typhi*	IgM IFA	Phetsouvanh *et al.* (2009),[17] Coleman *et al.* (2002)<br><br>Jiang *et al.* (2012)[21]<br><br>Henry *et al.* (2007)[23]
O. tsutsugamushi	------------	Culture of EDTA anti-coagulated buffy coat	TaqMan real-time PCR for 47 kDa gene on buffy coat	IgM IFA	Jiang *et al.* (2004)[21]
SFG *Rickettsia* spp.	------------	-------	Nested conventional PCR assays targeting the 17-kDa, *gltA*, *ompB*, *ompA*, and *sca4* genes. If a positive amplicon was produced, DNA sequencing was performed	---------	Jiang *et al.* (2005)[24]<br><br>Jiang *et al.* (2012)[22]
Dengue and JEV	--------------	-------	TaqMan real-time RT-PCR for dengue	PanBio ELISA for dengue NS1, IgM & IgG and JEV IgM	Jacobson *et al.* (2007) Leparc-Goffart *et al.* (2009)[18]

SFG : spotted fever group

A. Sample transport

Samples were packed in plastic screw capped tubes with paper wadding, in double skinned locked metal boxes (Blacksell *et al.* 2006). LNT specimens were transported at ambient temperature by flight (~90 minutes) or by bus (~24 h) to Vientiane and SV specimens by bus (~24 h). Temperature loggers (TinyTag Transit-2 TG0050, Gemini Data Loggers (UK)) were kept in each metal box from October 2009, to record temperature every 2 hours. The mean (+/- 2SD; range) temperatures measured within the transport boxes (for 15 months) were 25 (15.6-34.4; 11.0-44.2)^{o}C for LNT and 26 (18.8-33.2; 14.1-38.5)^{o}C for SV (T test, $P<0.0001$).

B. Malaria slides and RDTs

Giemsa-stained malaria smears and pLDH-based ICT Malaria Combo Cassette Test (ICT Diagnostics, Cape Town, South Africa) were performed for all patients. Slides were re-checked by a Centre for Malariology, Parasitology & Entomology (CMPE) World Health Organization (WHO)-accredited Level 1 microscopist, blinded to the results from the provincial hospitals.

C. Blood cultures

Blood culture bottles (Pharmaceutical Factory No. 2, Vientiane)[3] were weighed to estimate the volume of blood added to the bottles (specific gravity of blood assumed=1.06 (Trudnowski & Rico 1974)). Bottles from each batch were inoculated with reference organisms (*Pseudomonas aeruginosa*, *S. pneumoniae* and *H. influenzae*) to check for their growth and without inoculation, for no growth. The Mahosot laboratory participates in the UK NEQAS General Bacteriology and Antimicrobial Susceptibility Testing scheme. Positive cultures were identified using conventional techniques[3] and antibiotic susceptibility determined by disc diffusion using Clinical and Laboratory Standards Institute (CLSI) criteria.[14] True bacteraemia and blood culture contaminants were defined using conventional criteria.3 Blood culture results were reported as soon as any clinically relevant

information on bacterial growth was available and may therefore have influenced patient management.

D. Dengue/JEV ELISAs

The following Panbio Inc. (now Inverness Inc.) ELISA kits were used to investigate dengue and JEV infection (Jacobson *et al*. 2007, Moore *et al*. 2012).[10]

a. Japanese encephalitis - Dengue IgM Combo (E-JED01C) – detects and distinguishes IgM against dengue and JEV

b. Dengue IgG Capture (E-DEN02G) – detects high level of anti-dengue IgG in acute secondary dengue infection. We refer to this kit as anti-dengue HL-IgG ELISA.

c. Dengue IgG indirect ELISA (E-DEN01G) permits detection of low level of anti-dengue IgG including IgG from past exposure. We refer to this kit as anti-dengue LL-IgG ELISA.

d. Dengue Early ELISA (E-DEN01P) – detects dengue non-structural protein-1 (NS1). Has high specificity plus, during the first ~ 5 days of illness high sensitivity.

These were used, according to the kit instructions, to determine the following categories:

Dengue or JEV IgM positive

Dengue NS1 positive

HL-IgG positive

LL-IgG positive

In collaboration with PanBio Inc. a schema was developed for the interpretation of these kits when used together (see Supplementary Material-2). There is good agreement between the Japanese encephalitis - Dengue IgM Combo ELISA and the reference AFRIMS dengue/JEV ELISA.[10]

E. Immunofluorescence Assays (IFA) for antibodies against *O. tsutsugamushi* and *R. typhi*

The procedure followed that of Phetsouvanh *et al.* (2009).[17] Dried blood spots were collected onto Proteinsaver filter paper strips and transported and stored in sealed plastic bags, containing silica gel crystals. In Vientiane a cardpunch was used to cut 6 mm diameter discs from the blood-impregnated filter paper blood spots, halfway between the center and the edge of the blood spot. These were eluted overnight in 250µl autoclaved PBS at 37°C. Saturated discs were equivalent to a 1/25 dilution of serum. The eluted samples were serially diluted in sterile PBS to 1/12,800 and added to IFA slides (coated with antigen of *O. tsutsugamushi* strains Karp, Kato and Gilliam serotypes) and *R. typhi* strain (Wilmington) (Australian Rickettsial Reference Laboratory) and incubated in a moist chamber at 37°C for 1 hour. The slides were then washed three times for 5 minutes each with autoclaved PBS. After washing and drying, the slides were treated with specific fluorescein isothiocyanate conjugated goat anti-human gamma chain immunoglobulin (Sigma Aldrich, Germany), incubated for 30 min at 37°C, washed three times for 5 minutes each with autoclaved PBS, and mounted in buffered glycerol (90% v/v glycerol and 10% PBS). The IFA slides were read on a Nikon ECLIPSE E600 microscope (Nikon Co., Japan) by RP & TT. The end point of each IFA titer was defined as the lowest serum concentration demonstrating definite fluorescence. One IFA read the majority of the slides. A positive result was defined as an IgM or IgG titer ≥ 1/400.[17] However, whether this is the correct cutoff titre for the Lao situation is uncertain.

F. Extraction of Nucleic Acids

DNA

Approximately 500 DNA extractions from buffy coat samples were performed with FlexiGene DNA kits (Qiagen) according to the manufacturer's instructions. The starting volumes varied between 100-500µl of buffy coat.

The remaining samples (whole blood, red cell pellet and buffy coat) were extracted with QIAamp DNA Mini kits (Qiagen). These spin column-based kits have been used for the rest of the study, according to the manufacturer's instructions. The lysis incubation time at 56ºC was increased from 10 min to 1 hour such as suggested by Qiagen technical support. The starting volume was 200µl while the final elution volume was 100µl.

Measurements with a NanoDrop spectrophotometer (Thermo Scientific) were done after each extraction to assess the concentration and the purity of the extracted DNA. The extracts were then divided in 2 aliquots (for subsequent PCR duplicates) and kept at -80°C.

RNA

The first 40 serum samples were extracted with the EZ1 Virus Mini Kit v2.0 , using a BioRobot EZ1 Workstation (Qiagen). All the remaining RNA extractions were performed with the manual QIAamp Viral RNA Mini kit (Qiagen) from serum samples. For this kit, the starting volume was 140µl while the final elution volume was 80µl. The extracts were then divided in 2 aliquots (for subsequent PCR duplicates) and kept at -80°C.

For each RNA extraction, a fixed quantity of RNA bacteriophage (the MS2 bacteriophage) was added to the sample as an internal control. Once the extraction was finished, a one step real time RT-PCR (Reverse Transcription-PCR) using MS2 specific primers and probe was then performed on a random selection of RNA extract samples. This was done to control for RNA extraction and to check for the presence of PCR inhibitors according to the technique developed by Ninove *et al.* (2011).

Each PCR was done in duplicate. A third PCR was performed on samples with discordant duplicates.

G. Dengue PCR

This assay is a single step TaqMan real-time reverse transcriptase PCR performed with the SuperScript III Platinum One-Step qRT-PCR kit (Invitrogen), using 5µl of RNA extract in a 25µl reaction volume. Primers and probes follow Leparc-Goffart *et al*. (2009)[18] and the technique used was replicated from that used at the virology laboratory at La Timone Hospital, Marseille, where it was developed. The primers and probe for the Pan-Dengue PCR were designed to allow detection of the 3'NC region. Those for serotype specific detection are in the capsid region except primers and probes for the Dengue 2 system, which are located on the overlapping 5'NC and capsid region. Synthetic RNA was used as positive control.

H. Malaria nested conventional PCR

The nested conventional PCR assay of Snounou & Singh (2002)[19] was used. Two µl of DNA extract from whole blood (or from red cell pellet if whole blood was not available) served as template in a 22 µl reaction final volume with the Taq DNA Polymerase recombinant kit (Invitrogen). Each PCR was performed in duplicate, and a sample that was positive was subsequently tested for *P. falciparum* and *P. vivax*. All PCR products were visualized on 2% agarose gel (in 0.5X TBE buffer) with ethidium bromide.

I. *Leptospira* spp. real-time PCR

This assay is a TaqMan real-time PCR using 5µl of extract from whole blood (or 2µl of extract from buffy coat if whole blood was not available) in a 20µl reaction volume. The assay is based on Thaipadungpanit *et al*. (2011),[20] which detects the *Leptospira rrs* gene (16S rRNA). Each run contained duplicate low-positive standard dilutions, based on calculated genomic equivalents (GE) of genomic DNA extracted from *L. interrogans* serovar Lai strain. One GE/µl of genomic DNA stock was estimated based on a genomic size of 4.659 Mb for *L. interrogans* serovar Lai strain, and low-positive dilutions over 4 orders of magnitude (1 to 1 x 10^3 (GE/reaction)) served as positive controls. A one copy/µl standard was detected in each run. An important consideration is that the PCR of Thaipadungpanit *et al*. (2011)[20] will, as the target for the *rrs* assay is ubiquitous amongst *Leptospira*, detect pathogenic, intermediate and non-pathogenic groups. However, unless samples or experiments were contaminated we would not expect non-pathogenic *Leptospira* in patients' blood.

J. *Orientia tsutsugamushi* real-time PCR

This probe-based real-time TaqMan PCR was based on that described by Jiang *et al*. (2004).[21] The primers and probe sequences are based on the 47-kDa outer membrane protein gene of the Karp, Kato, Gilliam and Boryong *O. tsutsugamushi* strains. One µl of DNA extract from EDTA buffy coat was used in a 25µl reaction with the Platinum Quantitative PCR SuperMix-UDG (Invitrogen) kits. The kit's indications were used and the PCR profile adjusted accordingly. Each run contained duplicate low-positive standard dilutions (linearized pGEM plasmids), down to one copy/µl and only runs detecting $\geq$10 copies/ µl standards were considered positive.

K. *Rickettsia* genus real-time PCR

This assay is a TaqMan real-time qPCR using 1µl of buffy coat extract as DNA template, in a 25µl reaction volume. It is based on Jiang *et al*. (2004, 2012)[21,22],and targets the 17kDa gene. The primers and probe sequences were selected from a 17-kDa gene consensus sequence derived from 21 species of *Rickettsia*. The Platinum® Quantitative PCR SuperMix-UDG kit (Invitrogen) was used. The DNA from two reference *Rickettsia* species, *R. typhi* and *R. honeii*, were used as positive controls.

L. *R.typhi* real-time PCR

This assay is based on Henry *et al*. (2007)[23] and detects a unique sequence in the outer membrane protein B gene (*ompB*) of *R. typhi*. It is a TaqMan real-time PCR using 1µl of buffy coat extract as a template, in a 25µl reaction volume, with the Platinum Quantitative PCR SuperMix-UDG (Invitrogen) kits. Probe and primer concentrations were optimized. A third PCR was performed on samples with discordant duplicates. Each run contained duplicate low-positive standard dilutions (linearized pGEM plasmids), down to one copy/µl and only runs detecting $\geq$10 copies/ µl standards were considered positive.

M. Determination of Spotted Fever Group *Rickettsia* species real-time PCR

Rickettsia genus 17kDa real-time PCR positive and *ompB* (*R. typhi*) real-time PCR negative samples were considered *Rickettsia* spp. positive. These samples were further analysed by a panel of nested conventional PCR assays targeting the 17-kDa, *gltA, ompB, ompA,* and *sca4* genes, as previously described (Jiang *et al.* 2005, 2012).[22,24] PCR positive amplicons underwent DNA sequencing by Macrogen, Seoul, South Korea, using BigDyeTM terminator cycling conditions on an automated ABI

model 3730XL nucleotide sequencer (Applied Biosystems, Foster City, CA, USA). The DNA sequences were analyzed for similarity of nucleotide sequences deposited on GenBank (via BLAST search).

N. Rickettsial Culture

Rickettsial culture was performed in the BSL3 Laboratory by inoculation of buffy coat onto Vero and L929 cells with incubation at 35°C in 5% CO_2 for 6-8 weeks.[15] Identity of rickettsial growth was confirmed by IFA and PCR. This work was started in August 2009 and therefore the data presented here represent 16 months of rickettsial culture.

O. Leptospiral Culture

Leptospire culture was performed using the clot remaining after centrifugation of the blood sample, collected in the tube containing no anticoagulant tube, after serum was removed, as described by Wuthiekanun *et al.* (2007).[16] It is important to note that such clot is not the optimal sample for leptospiral culture[16] but it was the only sample type available. This work was started in August 2009 and therefore the data presented here represent 16 months of leptospiral culture.

Culture of leptospires from clot was performed using 3 ml of Ellinghausen, McCullough, Johnson and Harris (EMJH) medium supplemented with 3% rabbit serum and 0.1% agarose in 5-ml sterile plastic flat-based screw-cap tubes (Sterilin, Barloworld Scientific Ltd., United Kingdom). Three milliliters of EMJH was added to the blood clot remaining after centrifugation of ~ 5 ml whole blood, and removal of serum, using a sterile pipette and left overnight at room temperature. The next morning, the supernatant was transferred into a new 5-ml tube and incubated at Lao room temperature (~ 25^{0}C) for 12 weeks. Leptospires were identified by dark-field microscopy at x200 magnification.[16]

P. Leptospiral Microscopic Agglutination Tests (MAT)

MATs were performed at the WHO/AO/OIE Collaborating Centre for Reference & Research on Leptospirosis, Queensland Health Forensic and Scientific Services, Coopers Plains, Queensland, Australia as described in Syhavong *et al.* (2010).[9] Leptospiral MATs were regarded as positive if serum showed a titer of ≥1:400, or if paired sera demonstrated a four-fold rise. It is important to note that, in Thailand, the MAT is a poor predictor of the infecting serovar (Smythe *et al.* 2009).

Q. Influenza real-time PCR

Influenza PCR was only performed for patients from LNT Hospital during the period of this study, but has since started at SV. Nasopharyngeal swab and/or oropharyngeal swab (BD Universal Viral Transport Medium, Beckton Dickinson, Maryland, USA) were collected from June 2010 to December 2010. Mahosot Hospital sent the swabs plus copies of CRFs to NCLE as soon as possible after arrival in Vientiane. The testing strategy involved typing for influenza A and B viruses. If influenza A was positive, then sub-typing for H1, H3, and pdm H1N1 was performed. If these were all negative, sub-typing continued for H5. Swab specimens were extracted by using the QIAamp Viral RNA mini Kit (QIAGEN Inc.). The SuperScript III Platinum one-step quantitative RT-PCR system (Invitrogen, Carlsbad, CA, USA) was used for single-step real-time reverse transcription PCR according to US CDC protocol.[25] The primers and probes for the influenza virus (H1N1, H3N2, pandemic H1N1 2009, H5N1 and Influenza B) were derived by US-CDC to detect a fragment of gene M (specific of Influenza virus type A), gene NS (specific of Influenza virus type B) and gene HA (specific of Influenza virus subtype H1, H3, pandemic H1N1 2009 and H5).[25]

R. C-reactive protein

Serum C-reactive protein (CRP) was assayed in Institute Pasteur (IP) in Cambodia using latex beads immunoturbidimetric method on Integra 400 (Roche Diagnostics, Indianapolis, Ind., USA), on admission sera from 1,120 patients. The kit instructions give the expected upper limit as <5 mg/L (see http://www.www.roche-diagnostics.cz%2Fdownload%2Fcent%2Fintegra%2FMethod_manual%2F04_Specific%2520proteins%2FCRPLX%2520%2520V2_en.pdf&rct=j&q=Roche%20Diagnostics%20CRP%20Integra%20instructions&ei=nvGPTrfDMKaiAen39H0DQ&usg=AFQjCNEHHoNjsEvKG1X5enJTgCm2R2aRUw&cad=rja).

S. Concordance

For the PCR assays the concordance between duplicate runs was high (Supplementary Material 3).

T. Serum C-reactive protein (CRP) was assayed at the Institut Pasteur, Cambodia, using latex bead immunoturbidimetry (Integra 400, Roche Diagnostics, Indianapolis, USA), on admission sera from 1,120 patients with sera remaining (reference upper limit <5 mg/L).

W. Specimen exchange

In order to check the comparability of malaria PCR results, samples were exchanged between Institute Pasteur, Cambodia (IPC) and LOMWRU. Of 16 DNA extracts of red blood cell pellets sent to IPC for malaria PCR, there was 100% concordance between the IPC and LOMWRU results. IPC used a nested PCR approach targeting the *cytb* gene followed by sequencing (Steenkeste *et al.* 2009). Of 18 RNA extracts from sera sent to IP from LOMWRU, 17/18 (94%) concordance was

found with one serum, dengue PCR positive in LOMWRU, negative in IPC. IPC used a multiplex nested reverse transcriptase PCR for dengue diagnosis (Lanciotti *et al.* 1992). Importantly, as we did not send primary specimen types these sample exchanges do not examine differences in nucleic acid extraction.

Supplementary Material – 2

Algorithm to determine each patient's infection status according to the results from JE/Dengue IgM Combo ELISA, dengue IgG capture ELISA (HL-IgG), dengue IgG indirect ELISA (LL-IgG) and dengue Early ELISA (detects NS1).

The table below was used to interpret the results of the 3 ELISA kits JEV/Dengue IgM Combo, dengue IgG capture and dengue Early for distinguishing dengue, JEV and flavivirus infections.

For patients with anti-dengue IgM or HL-IgG positive on admission but who were negative in both kits using convalescent sera (when taken within 30 days of admission serum), after repetition, we concluded that they did not have dengue infection and assumed that the admission results represented a nonspecific immune response.

A FALSE result can be a low positive. In case of FALSE IgM or HL-IgG result in admission with negative or still FALSE result in convalescent sample, the infection status is "negative" (a low positive in admission should have been positive in convalescent sample). If IgM or HL-IgG are negative in admission sample then become FALSE in convalescent or in the case they are FALSE in admission with no convalescent sample the infection status stay "uncertain".

Primary and secondary dengue were distinguished according to the following:

Primary dengue: LL-IgG negative in acute sample.

Secondary dengue:

- Admission: IgG positive + IgM negative, convalescent: IgM positive
- Admission: IgG positive + NS1 positive in patient with less than 5 days of fever
- Admission: LL-IgG positive + HL-IgG negative + IgM negative, convalescent: HL-IgG positive + IgM negative.

In other cases, like IgM positive and IgG positive in admission, because we are not sure that is an acute dengue, it could be a primary or a secondary, we reported as "unknown" for primary or secondary.

Interpretation table:

Admission				Convalescent		Action	infection status
**IgM**	**NS1**	**HL-IgG**	**acute result**	**IgM**	**HL-IgG**		
N	N	N	negative	N	N	ok	N
N	N	N	negative	dengue	N	ok	dengue
N	N	N	negative	N	P	ok	dengue
N	N	N	negative	dengue	P	ok	dengue
N	N	N	negative	JEV	N	ok	JEV
N	N	P	dengue	N	P	ok	dengue
N	N	P	dengue	Dengue	P	ok	dengue

N	P	N	dengue	N	N	Check interval between Adm and Conv samples	dengue
N	P	N	dengue	dengue	N	ok	dengue
N	P	N	dengue	N	P	ok	dengue
N	P	N	dengue	dengue	P	ok	dengue
N	P	P	dengue	N	P	ok	dengue
N	P	P	dengue	dengue	P	ok	dengue
dengue	N	N	dengue	dengue	N	ok	dengue
dengue	N	N	dengue	dengue	P	ok	dengue
dengue	P	N	dengue	dengue	N	ok	dengue
dengue	P	N	dengue	dengue	P	ok	dengue
dengue	P	P	dengue	dengue	P	ok	dengue
dengue	N	P	dengue	Dengue	P	ok	dengue
JEV	N	N	JEV	JEV	N	ok	JEV
dengue	N	N	dengue	N	P	ok	dengue
dengue	P	N	dengue	N	P	ok	dengue
dengue	N	P	dengue	N	P	ok	dengue
dengue	P	P	dengue	N	P	ok	dengue
N	N	N	N	JEV	P	repeat	uncertain
N	N	P	dengue	N	N	repeat	uncertain
N	N	P	dengue	dengue	N	repeat	uncertain
dengue	N	N	dengue	N	N	repeat	uncertain
dengue	N	N	dengue	JEV	N	repeat	flavivirus
dengue	N	N	dengue	JEV	P	repeat	flavivirus
N	P	P	dengue	N	N	repeat	uncertain
N	P	P	dengue	Dengue	N	repeat	uncertain
dengue	P	N	dengue	N	N	repeat	uncertain
dengue	P	N	dengue	JEV	N	repeat	dengue
dengue	P	N	dengue	JEV	P	repeat	dengue
dengue	P	P	dengue	N	N	repeat	uncertain
dengue	P	P	dengue	dengue	N	repeat	uncertain
dengue	P	P	dengue	JEV	N	repeat	dengue
dengue	P	P	dengue	JEV	P	repeat	dengue
dengue	N	P	dengue	N	N	repeat	uncertain
dengue	N	P	dengue	dengue	N	repeat	uncertain
dengue	N	P	dengue	JEV	N	repeat	flavivirus
dengue	N	P	dengue	JEV	P	repeat	flavivirus
JEV	N	N	JEV	N	N	repeat	uncertain
JEV	N	N	JEV	dengue	N	repeat	flavivirus
JEV	N	N	JEV	N	P	repeat	flavivirus
JEV	N	N	JEV	dengue	P	repeat	flavivirus
JEV	N	N	JEV	JEV	P	repeat	flavivirus

JEV	P	N	uncertain	N	N	repeat	uncertain
JEV	P	N	uncertain	dengue	N	repeat	dengue
JEV	P	N	uncertain	N	P	repeat	dengue
JEV	P	N	uncertain	dengue	P	repeat	dengue
JEV	P	N	uncertain	JEV	N	repeat	dengue
JEV	P	N	uncertain	JEV	P	repeat	dengue
JEV	N	P	uncertain	N	N	repeat	uncertain
JEV	N	P	uncertain	dengue	N	repeat	flavivirus
JEV	N	P	uncertain	N	P	repeat	flavivirus
JEV	N	P	uncertain	dengue	P	repeat	flavivirus
JEV	N	P	uncertain	JEV	N	repeat	flavivirus
JEV	N	P	uncertain	JEV	P	repeat	flavivirus
JEV	P	P	uncertain	N	N	repeat	uncertain
JEV	P	P	uncertain	dengue	N	repeat	uncertain
JEV	P	P	uncertain	N	P	repeat	dengue
JEV	P	P	uncertain	dengue	P	repeat	dengue
JEV	P	P	uncertain	JEV	N	repeat	uncertain
JEV	P	P	uncertain	JEV	P	repeat	dengue
N	N	P	dengue	JEV	N	repeat	uncertain
N	P	P	dengue	JEV	P	repeat	dengue
N	P	P	dengue	JEV	N	repeat	dengue
N	P	N	dengue	JEV	P	repeat	dengue
N	P	N	dengue	JEV	N	repeat	dengue
N	N	P	dengue	JEV	P	repeat	flavivirus

Supplementary Material-3. Concordance between the first and the second runs of PCR assays

Assays					
Malaria	Leptospirosis	Dengue	*O. tsutsugamushi*	*Rickettsia* spp.	*R.typhi*
1,835/1,836 (99.9%)	1,860/1,877 (99.1%)	1,878/1,898 (98.9%)	1,835/1,848 (99.2%)	1,831/1,844 (99.2%)	12/17 (70.5%)

Supplementary Material-4. Demographic and clinical features of NMFI patients recruited at Luang Namtha and Salavan Provincial Hospitals. Data shown as number (%) or mean (95%CI) except + median (range). P values for each site, for comparison of patients admitted and not admitted given. In first column * = significantly different between Luang Namtha and Salavan (P<0.05).

Variable	Luang Namtha Provincial Hospital				Salavan Provincial Hospital			
	**Total (n = 1,390)**	**Patients admitted (n=389, 28%)**	**Patients not admitted (n=1,001, 72%)**	***P*-value**	**Total (n = 548)**	**Patients admitted (n = 468, 85.4%)**	**Patients not admitted (n = 80, 14.6%)**	***P*-value**
**Demography**								
Age/years +*	19 (5-49)	16 (5-48)	20 (5-49)	<0.001	20 (5-49)	20 (5-49)	22.5 (6-49)	0.39
Age ≤ 15 years*	540/1,390 (38.8)	191/389 (49)	349/1,001 (35)	<0.001	183/548 (33.3)	158/468 (33.7)	25/80 (31.2)	0.66
Male*	782/1,390 (56.2)	207/389 (53)	575/1,001 (57)	0.154	348/548 (63.5)	296/468 (63.2)	52/80 (65)	0.764
Weight/kg +	47 (10 – 85) n = 722	34 (12 – 81) n = 235	50 (10 – 85) n = 487	<0.001	46 (10 – 86) n = 419	46 (10 – 86) n = 397	50 (22 – 70) n = 22	0.10
Height/cm +	155 (85-180) n = 518	148 (85-174) n = 141	158 (85-180) n = 377	<0.001	155 (100-170) n = 277	155 (100-170) n = 256	160 (140-166) n = 21	0.03
**Symptoms**								
Days ill +*	4 (1 – 150)	5 (1 – 60)	4 (1 – 150)	<0.001	5 (1 – 730)	5 (1 – 730)	5 (1 – 30)	0.06
Days febrile +*	3 (1 – 8)	4 (1 – 8)	3 (1 – 8)	<0.001	5 (1 – 8)	5 (1 – 8)	4 (1 – 8)	0.17
Headache*	777/825 (94)	275/308 (89)	502/517 (97)	<0.001	437/500 (87.4)	387/443 (87.3)	50/57 (87.7)	0.93
Myalgia*	707/819 (86)	225/302 (75)	482/517 (93)	<0.001	292/445 (65.6)	270/413 (65.3)	22/32 (68.7)	0.69
Arthralgia	508/821 (61.8)	91/304 (30)	417/517 (81)	<0.001	286/441 (64.8)	272/415 (65.5)	14/26 (53.8)	0.22
Retro-orbital	116/808 (14.3)	37/294 (13)	79/514 (15)	0.28	41/425 (9.6)	39/400 (9.7)	2/25 (8)	1

pain*								
Back pain*	550/820 (67)	159/304 (52)	391/516 (76)	<0.001	99/436 (22.7)	92/409 (22.4)	7/27 (25.9)	0.68
Nausea*	388/822 (47.2)	194/306 (63)	194/516 (38)	<0.001	125/438 (28.5)	118/410 (28.7)	7/28 (25)	0.66
Vomiting*	455/1384 (32.8)	213/385 (55)	242/999 (24)	<0.001	142/536 (26.4)	119/456 (26.1)	23/80 (28.7)	0.62
Diarrhoea*	215/1,384 (15.5)	91/385 (23.7)	124/999 (12)	<0.001	28/537 (5.2)	23/457 (5)	5/80 (6.2)	0.58
Abdominal pain*	219/817 (26.8)	116/301 (39)	103/516 (20)	<0.001	25/438 (5.7)	23/405 (5.6)	2/33 (6)	1
Constipation*	55/822 (6.6)	48/307 (16)	7/515 (1)	<0.001	12/433 (2.7)	12/407 (2.9)	0/26	1.00
Dysuria	53/1379 (3.8)	14/383 (4)	39/996 (4)	0.822	12/532 (2.2)	9/452 (1.9)	3/80 (3.7)	0.40
Cough*	501/1,382 (36.2)	142/387 (37)	359/1,001 (36)	0.77	94/539 (17.4)	77/459 (16.7)	17/80 (21.2)	0.33
Sputum*	119/822 (14.4)	37/308 (12)	82/514 (16)	0.120	21/432 (5)	20/406 (4.9)	1/26 (3.8)	1.00
Dyspnoea	49/822 (5.9)	26/307 (8)	23/515 (4)	0.019	18/432 (4.1)	15/406 (3.6)	3/26 (11.5)	0.08
Sore throat*	393/1,380 (28.4)	97/381 (25)	296/999 (30)	0.125	82/535 (15.3)	65/455 (14.2)	17/80 (21.2)	0.11
Runny nose*	204/1,202 (16.9)	24/232 (10)	180/970 (19)	0.003	8/283 (2.8)	3/212 (1.4)	5/71 (7)	0.02
Ear pain	8/1,189 (0.6)	1/227 (0.4)	7/962 (0.7)	0.634	2/265 (0.75)	1/199 (0.5)	1/66 (1.5)	0.43
Hearing loss	10/813 (1.2)	8/297 (3)	2/516 (0.4)	0.004	1/409 (0.2)	1/386 (0.2)	0/23	1.00
**Signs**								
Temperature/^{0}C +	38.5 (36 – 41) n = 1,379	38.6 (36 – 41) n = 380	38.5 (36 – 40.7) n =999	<0.001	38.5 (36.5 – 41) n = 533	38.5 (36.5 – 41) n = 456	38 (37.5 – 40.2) n = 77	<0.001

Pulse/min	92.4 (91.3 – 93.5) n = 811	101 (99-103) n = 300	87 (86.2 – 89) n = 511	<0.001	92.3 (90.9 – 93.7) n = 430	92.7 (91.2 – 94.1) n = 408	85 (81.9 – 88.1) n = 22	0.01
Blood pressure systolic/mmHg*	107 (106.1-108) n = 713	105.3 (103.1-107.4) n = 265	108.1(107.2-109) n = 448	0.006	104.5 (103.3-105.6) n = 426	104.3 (103.1-105.5) n = 404	107.7 (103.2-112.2) n = 22	0.21
Blood pressure diastolic/mmHg *	65.9 (65.2 – 66.6) n = 710	64.3 (63 – 66) n = 263	67 (66 – 68) n = 447	0.001	69.5 (68.6 – 70.3) n = 426	69.4 (68.5 – 70.3) n = 404	70.9 (67.5 – 74.2) n = 22	0.46
Respiratory, rate/min *	22.5 (22.3 – 22.8) n = 784	24 (23 – 24) n = 284	22 (21.8 – 22.2) n = 500	<0.001	24.3 (23.9 – 24.7) n = 382	24.3 (23.9 – 24.8) n = 360	24.1 (22.0 – 26.2) n = 22	0.81
GCS+	15 (3 – 15) n = 794	15 (3 – 15) n = 297	15 (15 – 15) n = 497	<0.001	15 (5 – 15), n = 363	15 (5 – 15) n = 340	15 (15-15) n = 23	0.43
GCS <15	36/794 (4.5)	36/297 (12)	0/497 (0)	<0.001	9/363 (2.4)	9/340 (2.6)	0/22 (0)	1
Jaundice*	31/813 (3.8)	20/300 (7)	11/513 (2)	0.001	33/418 (7.8)	32/396 (8)	1/22(4.5)	1
Rash*	75/1,320 (5.6)	35/371 (9)	40/949 (4)	<0.001	11/513 (2.1)	8/437(1.8)	3/76 (3.9)	0.21
Eschar*	19/811 (2.3)	13/296 (4)	6/515 (1)	0.003	1/404 (0.2)	1/382 (0.2)	0/22	1.00
Cutaneous abscess*	2/801 (0.2)	1/290 (0.3)	1/511 (0.2)	1.00	17/419 (4)	15/396 (3.7)	2/23 (8.7)	0.23
Anaemia*	184/815 (22.5)	126/301 (42)	58/514 (11)	<0.001	43/419 (10.2)	42/397 (10.5)	1/22 (4.5)	0.71
Injected pharynx	94/811 (11.5)	50/295 (17)	44/516 (9)	<0.001	42/412 (10.1)	42/389 (10.8)	0/23	0.15
Conjunctival suffusion *	89/815 (10.9)	32/298 (11)	57/517 (11)	0.899	75/418 (17.9)	70/395 (17.7)	5/23 (21.7)	0.62
Septic arthritis*	3/804 (0.3)	3/294 (1)	0/510 (0)	0.049	9/416 (2.1)	8/394 (2)	1/22 (4.5)	0.39
Abdominal tenderness*	108/813 (13.2)	93/300 (31)	15/513 (3)	<0.001	30/422 (7.1)	28/399 (7)	2/23 (8.7)	0.67
Hepatomegaly	39/738 (5.2)	37/292 (13)	2/446 (0.5)	<0.001	16/415 (3.8)	16/392 (4)	0/23	1.00

Splenomegaly*	28/737 (3.8)	26/292 (9)	2/445 (0.5)	<0.001	7/414 (1.6)	5/391 (1.2)	2/23 (8.7)	0.052
Abnormal chest	34/819 (4.1)	30/302 (10)	4/517 (0.8)	<0.001	21/423 (4.9)	19/400 (4.7)	2/23 (8.7)	0.31
Abnormal heart	28/815 (3.4)	25/298 (8)	3/517 (0.6)	<0.001	14/417 (3.3)	14/394 (3.5)	0/23	1.00
Lymphadenopathy*	218/824 (26.4)	147/303 (49)	71/521 (14)	<0.001	32/419 (7.6)	29/397 (7.3)	3/22 (13.6)	0.23
Seizure*	35/822 (4.2)	30/307 (10)	5/515 (1)	<0.001	6/431 (1.3)	6/405 (1.4)	0/26	1.00
Neck stiffness*	35/793 (4.4)	33/287 (12)	2/506 (0.4)	<0.001	2/406 (0.4)	2/384 (0.5)	0/22	1.00
Confusion*	46/820 (5.6)	44/305 (14)	2/515 (0.4)	<0.001	8/431 (1.8)	8/405 (1.9)	0/26	1.00
Drowsiness	63/819 (7.6)	56/303 (18)	7/516 (1)	<0.001	24/433 (5.5)	24/407 (5.9)	0/25	0.38
Acute encephalitis syndrome *	58/825 (7.0)	53/296 (18)	5/498 (1)	<0.001	13/432 (3.0)	13/406 (3.2)	0/26	1.00
Meningitis	51/773 (7.0)	46/282 (16)	5/491 (1)	<0.001	9/361 (2)	9/338 (3)	0/23	1.00
Tourniquet test positive*	8/275 (3)	7/156 (4)	1/119 (0.8)	0.14	18/259 (7)	16/243 (7)	2/16 (13)	0.307
**Investigations**								
Haematocrit/ % *	41.2 (40.4 – 41.9) n = 503	36.7 (35.4 – 38) n = 186	44 (43 – 45) n = 317	<0.001	38.2 (37.1 – 39.3) n = 155	38.3 (37.2 – 39.4) n = 153	32.3 n=2	0.22
WBC,/mm^3	9,594 (8,984- 10,144), n = 509	10,896 (9,808- 11,983), n = 187	8,791 (8,136- 9,447), n = 322	<0.001	9,594 (8,583- 10,606), n = 154	9,559 (8,535-10,583), n = 152	12,250 (9,073 – 15,426) n=2	0.55
Neutrophils,/%	68.7 (67.3 – 69.9) n = 508	71 (68 – 73) n = 187	68 (66 – 69) n = 321	0.027	68.2 n = 81	68.5 (65.0 – 72.0) n = 80	80 n=1	NA
Lymphocyte /mm^3*	25.8 (23.9 – 27.7) n = 149	26 (24 – 28) n = 140	23 (12 – 33) n = 9	0.400	21.4 (5-88) n=105	21.4 (5-88) n=105	NA	NA
Platelets/mm^3 *	219,980 (211,403-228,558), n = 400	237,113 (215,319-258,908), n = 97	214,496 (205,581-223,411), n =	0.026	246,704 n = 77	243,485 (224,915-262,054) n = 120	633,000 n=1	NA

			303					
C reactive protein (CRP)/mg/L	77.3 (69.7-84.8), n=815	115 (99-131) , n=294	56 (49-63), n=521	<0.001	73.6 (63.3-83.9) n=355	78 (66.7-89.8) n=302	47 (28.2-66.3) n=53	0.01
CRP ≥ 5 mg/L	681/815 (84%)	262/288 (91)	419/527 (80)	<0.001	277/355 (78)	241/302 (80)	36/53 (68)	0.054

The mean (95%CI) duration samples in metal transport boxes (December 2009-2010) were 18.0 (16.2-19.8), 16.3 (12.0-20.5) and 19.2 (18.1-20.2) hours for all samples, LNT and SV, respectively.

Supplementary Material-5. Overall diagnoses of the patients enrolled in the NMFI study, using all assays, for monopathogens (top) and more than one pathogen (below).

Data shown as number (%). Influenza diagnosis was only conducted on samples collected from LNT for 6 months.

Variable	All (n = 1,938)	Luang Namtha (n = 1,390)	Salavan (n = 548)	P value
No diagnosis	762/1,938 (39.3)	583/1,390 (41.9)	179/548 (32.7)	<0.0001
With diagnosis	1176/1,938 (60.7)	807/1,390 (58.1)	369/548 (67.3)	<0.0001
With mono pathogens	797/1,938 (41.1)	551/1,390 (39.6)	246/548 (44.9)	0.034
Dengue fever (Serology, NS1, PCR)	255/1,928 (13)	124/1,383 (9)	131/545 (24)	<0.0001
Scrub typhus (IFA, PCR, Culture)	197/1,934 (10.2)	134/1,386 (9.7)	63/548 (11.5)	0.231
Japanese encephalitis virus (Serology)	91/1,924 (4.7)	73/1,383 (5.3)	18/541 (3.3)	0.07
Leptospirosis (PCR, Culture, MAT)	86/1,934 (4.5)	77/1,389 (5.5)	9/545 (1.7)	<0.0001
Influenza (PCR)	85/358 (23.7)	85/358 (23.7)	-	-
Murine typhus (IFA, PCR)	36/1,919 (1.9)	29/1,371 (2.1)	7/548 (1.3)	0.222
Community acquired bacteraemia	29/1,938 (1.5)	21/1,390 (1.5)	8/548 (1.5)	1.0
Malaria * (RDT, Smear, PCR)	12/1,938 (0.6)	3/1,390 (0.2)	9/548 (1.6)	0.001
Undetermined *Rickettsia* spp. and *R. felis* (PCR)	5/1,849 (0.3)	4/1,320 (0.3)	1/529 (0.2)	1
Flavivirus infection (Serology, NS1, PCR)	1/1,926 (0.05)	1/1,383 (0.07)	0/543 (0)	-
Multiple diagnoses	376/1,938 (19.4)	256/1,390 (18.4)	120/548 (21.9)	0.081
With 2 pathogens	326/1,938 (16.8)	218/1,390 (15.7)	108/548 (19.7)	0.033
With more than 2 pathogens	50/1,938 (2.6)	38/1,390 (2.7)	12/548 (2.2)	0.496

* Of 25 patients who were diagnosed with malaria, 1/25 (4%) had co-infection of *Plasmodium falciparum* and *Plasmodium vivax*.

Of 1,983 patients who were included in this study, 802/1,983 (41%) had one pathogen detected, 320/1,938 (16.5%) had apparent co-infection with 2 different pathogens, 52/1,983 (3%) had 3 apparent pathogens, 2/1,983 (0.1%) had 4 different pathogens and 763/1,938 (39%) had no diagnosis.

Supplementary Material-6. Summary of results of microscopy, RDT and PCR assays for malaria diagnosis.
NB. The PCR assays do not distinguish sexual and asexual forms of *Plasmodium* species. The slide and RDT negative but PCR positive patients may therefore be carrying only gametocytes and malaria was not the cause of their presenting illness

**Variable**	**All (n = 1,938)**	**Luang Namtha (n = 1,390)**	**Salavan (n = 548)**	***P*-value**
Malaria positive (including all tests)	25/1,938 (1.3%)	4/1,390 (0.3%)	21/548 (4%)	< 0.001
Positive RDT for *P. falciparum*	10/1,938 (0.5%)	0/1,390 (0%)	10/548 (2%)	< 0.001
Positive RDT for *P. vivax*	1/1,938 (0.05%)	1/1,390 (0.07%)	0/548 (0%)	1.00
Positive Giemsa slide for *P. falciparum*	10/1,938 (0.5%)	0/1,390 (0%)	10/548 (2%)	< 0.001
Positive Giemsa slide for *P. vivax*	1/1,938 (0.05%)	1/1,390 (0.07%)	0/548 (0%)	1.00
Positive PCR for *Plasmodium* spp.	25/1,836 (1%)	4/1,308 (0.3%)	21/528 (4%)	<0.001
Positive PCR for *P. falciparum*	18/1,836 (0.9%)	0/1,308	18/528 (3%)	<0.001
Positive PCR for *P. vivax*	6/1,836 (0.3%)	4/1,308 (0.3%)	2/528 (0.4%)	1
Positive PCR for both *P. falciparum* & *P. vivax*	1/1,836 (0.05%)	0/1,308	1/528 (0.2%)	0.288

There was 100% concordance between RDTs and microscopy. The percentage of the concordance between Giemsa slide and PCR for *P. falciparum* and *P. vivax* were 1,826 /1,836 (99%) and 1,831/1,836 (99.7%), respectively. The percentage of the concordance between RDT and PCR for *P. falciparum* and *P. vivax* were 1,826 /1,836 (99%) and 1,831/1,836 (99.7%), respectively.

Supplementary Material-7. Estimated median (range) blood volume added to haemoculture bottles in relation to different bottle sizes and whether final report was of no growth or clinically significant positive haemoculture. The aim was to inject 2 ml blood/bottle from patients aged 5 to ≤ 15 years and 5 ml/bottle for patients aged 16-49 years. Specific gravity of blood assumed to be 1.06.

	**Bottle 1**	**Bottle 2**	*P value*
Bottles from patients aged 5 to ≤ 15 years	1.6 (0.03-8.6) ml	1.7 (0.01-16.1) ml	0.590
Bottles from patients aged 16-49 years	3.3 (0.04-12.3) ml	3.4 (0.03-12.1) ml	0.112

	**Haemoculture no growth**	**Clinically significant positive haemoculture**	
Age 5 to ≤ 15 years			
Bottle 1	1.5 (0.03-8.6) ml	1.5 (0.6-3.9) ml	0.750
Bottle 2	1.6 (0.01-16.1) ml	1.2 (0.2-4.5) ml	0.774
Age 16-49 years			
Bottle 1	3.1 (0.1-12.3) ml	2.7 (0.9-6) ml	0.391
Bottle 2	3.1 (0.03-12.1) ml	3.2 (0.8-8.2) ml	0.641

Supplementary Material - 8. Antibiotic drug susceptibilities, by disc diffusion, for clinically significant organisms grown from blood cultures. Susceptible/total tested

**Organism**	Ampicillin	Ceftriaxone	Chloramphenicol	Nalixidic acid	Co-trimoxazole	Ofloxacin	Cefalothin
**Luang Namtha**							
S. Typhi	30/30	30/30	30/30	29/30	28/28	---	---
Salmonella Group C	2/2	2/2	2/2	2/2	1/1	---	---
E. coli	1/2	1/2	2/2	---	2/2	2/2	0/2
Klebsiella pneumoniae	0/1	1/1	1/1	---	1/1	1/1	1/1
**Salavan**							
S. Typhi	8/8	8/8	8/8	8/8	7/7	---	---
E. coli	0/2	2/2	2/2	---	0/2	2/2	1/2
Klebsiella pneumoniae	0/1	1/1	1/1	---	1/1	1/1	1/1

Supplementary Material - 9. Serological, culture and PCR results for *Leptospira* and *Rickettsia* species

Variable	All (n = 1,938)	Luang Namtha (n = 1,390)	Salavan (n = 548)	*P*
Leptospira MAT				
MAT positive	84/1,276 (7%)	70/881 (8%)	14/395 (3.5%)	0.003
Titre >=400	15/1,276 (1.1%)	14/881 (2%)	1/395 (0.2%)	0.047
Titre >=4 fold rise	69/1,276 (5%)	56/881 (6%)	13/395 (3%)	0.025
Leptospira PCR	74/1,878 (4%)	64/1,345 (5%)	10/533 (2%)	0.004
Leptospira Culture	28/986 (3%)	27/713 (4%)	1/273 (0.4%)	0.002
Leptospira PCR and/or *Leptospira* Culture	83/1,889 (4%)	72/1,352 (5%)	11/537 (2%)	0.002
Leptospira PCR, *Leptospira* Culture and/or MAT with 4 fold rise	137/1,932 (7%)	115/1,387 (8%)	22/545 (4%)	0.001
Leptospirosis positive by any test	151/1,934 (8)	128/1,389 (9)	23/545 (4)	<0.001
**Rickettsial IFA**				
Rickettsial IFA (Titre ≥400)				
IgM against *O.tsutsugamushi*	407/1,918 (21%)	255/1,370 (19%)	152/548 (28%)	<0.001
IgG against *O.tsutsugamushi*	861/1,918 (45%)	600/1,370 (44%)	261/548 (48%)	0.128
IgM against *Rickettsia typhi*	135/1,918 (7%)	94/1,370 (7%)	41/548 (7%)	0.631
IgG against *Rickettsia typhi*	224/1,918 (12%)	180/1,370 (13%)	44/548 (8%)	0.002
**Rickettsial culture**				
O.tsutsugamushi isolated	29/881 (3%)	22/644 (3%)	7/237 (3%)	0.733
Rickettsia typhi isolated	0	0	0	
SFG *Rickettsia spp.* isolated	0	0	0	

Rickettsial PCR				
O.tsutsugamushi	167/1,848 (9%)	121/1,319 (9.2%)	46/529 (8.7%)	0.746
Rickettsia spp.	29/1,849 (1.6%)	22/1,320 (1.7%)	7/529 (1.3%)	0.591
Rickettsia typhi	12/1,849 (0.6%)	7/1,320 (0.5%)	5/529 (0.9%)	0.341
Rickettsia felis	2/1,849 (0.1%)	1/1,320 (0.08%)	1/529 (0.2%)	0.49
Undetermined *Rickettsia* spp.	15/1,849 (0.8%)	14/1,320 (1%)	1/529 (0.2%)	0.082
O.tsutsugamushi and/or *O.tsutsugamushi* isolated	170/1,871 (9%)	123/1,337 (9.1%)	47/534 (8.8%)	0.787
Scrub typhus positive by any test	447/1,934 (23)	316/1,386 (22.8%)	161/548 (29.4%)	0.002
Murine typhus positive by any test	145/1,919 (7.6%)	101/1,371 (7.4%)	44/548 (8%)	0.620
Typhus positive by any test	537/1,934 (27.8%)	365/1,386 (26.3%)	172/548 (31.4%)	0.025

Of those rickettsial PCR positive (either *O. tsutsugamushi*, *Rickettsia* spp., *R. typhi* or *R. felis*), one patient was positive for both *O. tsutsugamushi* and *Rickettsia* spp., one positive for both *O. tsutsugamushi* and *Rickettsia typhi* and one positive for both *O. tsutsugamushi* and *Rickettsia felis.*

MATs suggested that the most frequent serovars infecting patients with a diagnostic rise at LNT (n=57), based on the serovar with the maximum titre were hardjo (22), copenhageni (9), djasiman (6), hebdomadis (4), mwalok (4), bataviae (2), cynopteri (2), grippotyphosa (2), saxkoebing (1), javanica (1), celledoni (1), mini (1), autumnalis (1) and tarassovi (1). For those with a diagnostic rise at SV the serovars were (n=8) – hebdomadis (4), copenhageni (3) and mwalok (1). However, it is important to note that the MAT is a poor predictor of the infecting serovar (Smythe *et al.* 2009)

Supplementary Material - 10. Serology and PCR assays for dengue and JEV and influenza PCR.
Influenza diagnosis was only conducted on samples collected from LNT for 6 months.

Variable	All (n = 1,938)	Luang Namtha (n = 1,390)	Salavan (n = 548)	*P value*
**DENV-JE ELISA**				
DENV NS1 positive	123/1,902 (7)	34/1,374 (2.5)	89/528 (17)	<0.001
IgM against DENV positive in acute sera	281/1,875 (15)	151/1,351 (11)	130/524 (25)	<0.001
IgM against DENV positive in convalescent sera	283/1,357 (21)	140/949 (15)	143/408 (35)	<0.001
High level-IgG against DENV positive in acute sera	43/1,900 (2.2)	16/1,373 (1.2)	27/527 (5)	<0.001
High level-IgG against DENV positive in convalescent sera	75/1,382 (5)	19/966 (2)	56/416 (13)	<0.001
IgM against JEV positive in acute sera	152/1,875 (8)	121/1,351 (9)	31/524 (6)	0.03
IgM against JEV positive in convalescent sera	115/1357 (8)	99/949 (10)	16/408 (4)	<0.0001
**Dengue-JEV status according to PanBio ELISA interpretation**				
JEV Infection #	171/1,904 (9)	142/1,367 (10)	29/537 (5)	0.001
Dengue infection	409/1904 (21)	210/1,367 (15)	199/537 (37)	<0.001
Primary Dengue Infection	155/409 (38)	105/210 (50)	50/199 (25)	<0.001
Secondary Dengue Infection	70/409 (17)	35/210 (16)	36/199 (18)	0.704
Flavivirus infection	5/1,904 (0.26)	5/1,367 (0.37)	0/541 (0)	0.33
**Dengue and Influenza PCR**				
DENV All	143/1,904 (8)	34/1,368 (2)	109/536 (20)	<0.001

NS1 and/or PCR positive	182/1,927 (9)	54/1,382 (4)	128/545 (23)	<0.001
Total Influenza PCR positive	139/358 (39)	139/358 (39)	-	-
Influenza B	121/358 (34)	121/358 (34)	-	-
Influenza A/H3	10/358 (3)	10/358 (3)	-	-
Pandemic H1N1	8/358 (2)	8/358 (2)	-	-

Including those JEV ELISA positive, 3/171 were DENV PCR positive

Supplementary Material-11. Clinical features and diagnosis of patients with AES and meningitis. For definitions see Methods. Data shown as number (%) unless indicated

**Variable**	**Patients with both meningitis + AES (n =53)**	**Patients with AES (n = 71)**	**Patients with meningitis (n = 60)**
**Demography**			
Age, years, median (range)	17 (5 – 47)	16 (5 – 48)	18 (5 – 47)
Male	29 (55)	39 (55)	32 (53)
**Symptoms**			
Days ill, median (range)	5 (2 – 16)	5 (1 – 16)	5 (2 – 16)
Days febrile, median (range)	5 (1 – 8)	4 (1-8)	4 (1 – 8)
Headache	53/53 (100)	62/71 (87)	60/60 (100)
Myalgia	32/53 (60)	42/70 (60)	39/60 (65)
Arthralgia	19/53 (36)	23/70 (33)	35/60 (58)
Retro-orbital pain	4/51 (8)	4/68 (6)	6/58 (10)
Back pain	25/53 (47)	30/70 (43)	29/60 (48)
Nausea	34/53 (64)	40/71 (56)	37/60 (62)
Vomiting	28/53 (53)	35/71 (49)	29/60 (48)
Diarrhoea	7/53 (13)	9/71 (13)	9/60 (15)
Abdominal pain	18/53 (34)	23/71 (32)	20/60 (33)
Constipation	4/53 (8)	7/71 (10)	6/60 (10)
Dysuria	0/53	0/71	0/60
Cough	11/53 (21)	13/71 (18)	13/60 (22)
Sputum	3/53 (6)	3/71 (4)	5/60 (8)
Dyspnoea	8/53 (15)	12/71 (17)	9/60 (15)
Sore throat	11/52 (21)	12/69 (17)	13/59 (22)
Runny nose	0/28	0/37	1/32 (3)
Ear pain	0/28	0/37	0/32

Hearing loss	3/50 (6)	3/67 (4)	4/56 (7)
**Signs**			
Temperature,^{0}C, mean (95%CI)	38.5 (37.5 – 39.5)	38.7 (37.7 –39.7)	38.6 (37.6 – 39.6)
Pulse/min, mean (95%CI)	103.8 (77.7 – 129.9)	104.2 (79.3 – 129.1)	103.8 (77.9 – 129.7)
Blood pressure systolic, mmHg, mean (95%CI)	112 (88.9 – 135.1)	113.1 (90-136.2)	111.3 (89.4 – 133.2)
Blood pressure diastolic, mmHg, mean (95%CI)	66.7 (53.5 – 79.9)	67.7 (54.5 – 80.9)	66.4 (53.4 – 79.4)
Respiratory, rate/ min, median (range)	26 (18 – 50)	26 (18 – 50)	25 (18 – 50)
GCS, median (range)	11 (3 – 15), n = 48	12 (3 – 15), n = 65	12 (3 – 15), n = 54
Jaundice	3/52 (6)	4/69 (6)	4/58 (7)
Rash	3/52 (6)	3/69 (4)	3/58 (5)
Eschar	1/51 (2)	2/68 (3)	1/57 (2)
Cutaneous abscess	0/4	0/6	0/6
Anaemia	20/51 (39)	24/68 (35)	22/57 (39)
Injected pharynx	4/50 (8)	5/67 (7)	6/56 (11)
Conjunctival suffusion	3/52 (6)	3/69 (4)	4/59 (7)
Septic arthritis	0/50	0/67	0/56
Abdominal tenderness	9/52 (17)	12/69 (17)	10/59 (17)
Hepatomegaly	5/49 (10)	5/65 (8)	5/55 (9)
Splenomegaly	3/49 (6)	3/65 (5)	4/55 (7)
Abnormal chest	10/53 (19)	12/70 (17)	10/59 (17)
Abnormal heart	7/52 (13)	8/69 (12)	8/58 (14)
Lymphadenopathy	19/52 (37)	25/69 (36)	20/58 (34)
Seizure	33/53 (62)	41/71 (58)	33/60 (55)
Neck stiffness	32/51 (63)	33/68 (49)	36/58 (62)
Confusion	44/53 (83)	54/71 (76)	44/60 (73)
Drowsiness	49/53 (92)	60/71 (85)	51/60 (85)
**Investigation**			
Haematocrit, %, median (range)	38 (15 – 50), n = 29	38 (15 – 50), n = 39	38 (13 – 50), n = 34

WBC, /mm^3, median (range)	11,865 (1,970 – 23,000) n = 30	12,850 (1,970 – 36,400) n = 40	12,200 (1,970 – 30,500) n = 35
Neutrophils, %, median (range)	75 (16.9 – 92.2), n = 29	76 (16.9 – 92.2), n = 39	75 (16.9 – 92.2), n = 34
Lymphocyte, /mm^3, median (range)	25 (7.7 – 44), n = 18	23.5 (5.6 – 44), n = 24	25 (7.7 – 44), n = 20
Platelet, /mm^3, median (range)	180,000 (67,000–416,000) n = 17	189,500 (67,000–416,000) n = 20	180,000 (67,000–452,000) n = 19
CRP, mg/L, median (range)	32.3 (1 – 608.6), n = 30	45.9 (1 – 608.6), n = 42	53.1 (1 – 608.6), n = 33
Bacteraemia	2/49 (4)	2/66 (3)	2/55 (4)
JEV	21/50 (42)	29/67 (43)	21/57 (37)
Scrub typhus	0/50 (0)	2/67 (3)	0/57 (0)
Leptospirosis	2/53 (4)	3/71 (4)	2/60 (3)
Dengue	3/51 (6)	4/69 (6)	4/58 (7)
Malaria	1/53 (2)	1/71 (1)	1/60 (2)
Influenza	0/6 (0)	0/6 (0)	0/8 (0)
No diagnosis	21/53 (40)	27/71 (38)	26/60 (43)

Supplementary Material - 12. Combinations of conservative diagnoses for patients with evidence for infection with >2 pathogens (n=95 patients). Influenza diagnosis was only conducted on samples collected from LNT for 6 months.

	Malaria	Bacteraemia	Leptospirosis	Dengue	JEV	Scrub typhus	Murine typhus	Undetermined *Rickettsia* spp. infection and *R.felis*	Influenza
Malaria	22 #								
Bacteraemia	0	43#							
Leptospirosis	1	2	109 #						
Dengue	0	3	6	156 #					
JEV	1	2	8	0	112 #				
Scrub typhus	0	1	5	10	26	122 #			
Murine typhus	0	1	0	0		1	10 #		
Undetermined *Rickettsia* spp. infection and *R.felis*	0	1	1	2	1	1+	0	9#	
Influenza	0	0	3	4	16	1	0	0	115 #

* Three patients were positive for 3 pathogens (two patients with JEV + Scrub typhus + Leptospirosis+ and one patient with *R. felis* PCR positive + dengue PCR positive and *P.falciparum* PCR positive)
# Patients with only evidence for one pathogen using conservative diagnoses.

Supplementary Material-13. Overall diagnosis for patients with evidence for infection with >2 pathogens (n=374), using all assays. # patients with only evidence for one pathogen using all assays. Influenza diagnosis was only conducted on samples collected from LNT for 6 months. + without conclusive evidence for dengue or JEV

	Malaria	Bacteraemia	Leptospirosis	Dengue	JEV	Flavivirus infection +	Scrub typhus	Murine typhus	Undetermined *Rickettsia* spp. and *R. felis*	Influenza
Malaria	12#									
Bacteraemia	0	29#								
Leptospirosis	0	2	86#							
Dengue	6	10	16	255#						
JEV	1	2	8	0	91#					
Flavivirus infection	0	0	0	0	0	1#				
Scrub typhus	2	6	20	98	37	2	197#			
Murine typhus	0	1	3	7	3	0	56	36#		
Undetermined *Rickettsia* spp. and *R. felis*	0	1	1	3	1	0	2	0	5#	
Influenza	0	0	3	10	11	0	9	5	0	85#

Supplementary Material -14. The relationship between clinical features and diagnosis of patients recruited at Luang Namtha and Salavan Provincial Hospitals. For patients with only evidence for one pathogen using conservative diagnoses. For continuous variables with missing values, the superscript gives the sample size with data. For continuous variables we have used mean (95%CI) except for + when we use median (range).

	**No diagnosis** n=1,139	**Malaria** n=22	**Bacteraemia** n=43	**Leptospirosis** n=109	**Dengue** n=156	**JEV** n=112	**Scrub typhus** n=122	**Murine typhus** n=10	**Influenza** n=115
	**Number of positive/tested (%)**								
SV Hospital	301/548 (54.9)	18 (81.8)	13 (30.2)	16 (14.7)	115 (73.7)	22 (19.6)	36 (29.5)	4 (40)	-
LNT Hospital	838/1390 (60.3)	4 (18.2)	30 (69.7)	93 (85.3)	41 (26.3)	90 (80.4)	86 (70.5)	6 (60)	115 (100)
Age/years	20 (21.2-22.6)	24.9 (19.2-30.6)	20 (16.8-23.2)	18.2 (16.2-20.2)	21.8 (20.2-23.3)	22.8 (20.5-25.1)	23.3 (21.1-25.5)	25.7 (13.9-37.5	18.6 (16.8-20.5)
Aged 5-15 years	419 (36.8)	6 (27.3)	15 (65.1)	62 (56.9)	44 (28.2)	39 (34.8)	38/122 (31.1)	4 (40)	49 (42.6)
Aged 16-49 years	720 (63.2)	16 (72.7)	28 (34.9)	47 (43.1)	112 (71.8)	73 (65.2)	84 (68.9)	6 (60)	66 (57.4)
Male	653 (57.3)	17 (77.3)	24/42 (55.8)	79 (72.5)	98 (62.8)	66 (58.9)	60 (49.2)	6 (60)	60 (52.2)
**Symptoms**									
Days febrile	3.9 (3.9-4)	4.4 (3.7-5.1)	4.7 (4.1-5.3)	3.3 (3-3.6)	4.1 (3.8-4.4)	4.4 (4-4.7)	5.5 (5.2-5.8)	4.5 (3-6)	3.5 (3.3-3.8)
Headache	623/698 (89.3)	20/21 (95.2)	30/31 (96.8)	60/63 (95.2)	128/135(94.8)	73/83 (88)	81/87 (93.1)	6/7 (85.7)	112 (97.4)
Myalgia	490/664 (73.8)	14/20 (70)	25/31 (80.6)	51/60 (85)	98/121 (81)	63/80 (78.8)	70/84 (83.3)	5/7 (71.4)	109 (94.8)
Arthralgia	376/662 (56.8)	9/20 (45)	11/30 (36.7)	36/59 (61)	95/121 (78.5)	41/79 (51.9)	62/86 (72.1)	3/7 (42.9)	104 (90.4)
Retro-orbital pain	72/650 (11.1)	2/18 (11.1)	1/28 (3.6)	11/59 (18.6)	17/117 (14.5)	6/78 (7.7)	7/82 (8.5)	3/7 (42.9)	26/114 (22.8)
Back pain	327/662 (49.4)	6/20 (30)	17/30 (56.7)	31/59 (52.5)	41/118 (34.8)	38/79 (48.1)	48/86 (55.8)	62/7 (28.6)	92/114

									(80.7)
Vomiting	313/1136 (27.6)	7/21 (33.3)	19/42 (45.2)	58/108 (53.7)	45/151 (29.8)	39/111 (35.1)	34/120 (28.3)	6/10 (60)	31/114 (27.2)
Diarrhoea	146/1133 (12.9)	2/21 (9.5)	13 (30.2)	18 (16.5)	14/153 (9.2)	11 (9.8)	13/120 (10.8)	1 (10)	10/113 (8.9)
Abdominal pain	129/661 (19.5)	3/19 (15.8)	12/30 (40)	17/60 (28.3)	8/119 (6.7)	19/82 (23.2)	18/83 (21.7)	3/7 (42.9)	25/113 (22.1)
Constipation	36/661 (5.5)	0/19 (0)	2/31 (6.5)	3/59 (5.1)	3/119 (2.5)	4/80 (5)	10/84 (11.9)	0/7 (0)	2/113 (1.8)
Dysuria	48/1127 (4.3)	0/21 (0)	4/42 (9.5)	2 (1.8)	2/150 (1.3)	3 (2.7)	3/120 (2.5)	0 (0)	2/113 (1.8)
Cough	379/1133 (33.5)	4/21 (19.1)	11 (25.6)	18/108 (16.7)	21/153 (13.7)	27/111 (24.3)	33/121 (27.3)	3 (30)	63/113 (55.8)
Dyspnoea	44/662 (6.7)	0/19 (0)	3/31 (9.7)	2/59 (3.4)	2/118 (1.7)	6/80 (7.5)	5/84 (6)	0/7 (0)	3/113 (2.7)
Sore throat	298/1131 (26.4)	1/21 (4.8)	4/42 (9.5)	27 (24.8)	31/152 (20.4)	21/110 (19.1)	26/120 (21.7)	4 (40)	40/113 (35.4)
Running nose	141/899 (15.7)	0/9 (0)	3/27 (11.1)	5/89 (5.6)	8/97 (8.3)	11/88 (12.5)	7/78 (9)	1/4 (25)	26/109 (23.9)
Hearing loss	2/645 (0.3)	0/18 (0)	0/30 (0)	1/58 (1.7)	1/110 (0.9)	2/77 (2.6)	1/82 (1.2)	1/7 (14.3)	1/115 (0.9)
**Signs**									
Temperature ^{0}C	38.5 (38.4-38.6) 1121	38.5 (38.1-39) 21	38.9 (38.6-39.1) 42	38.9 (38.8-39.1)	38.6 (38.5-38.7)	38.6 (38.4-38.7) 109	38.8 (38.6-38.9) 121	38.5 (38.1-38.8) 9	38.5 (38.4-38.6)

Pulse/min	91.7 (90.5-92.9) 653	91.9 (84.3-99.6) 19	97.6 (91.4-103.9) 30	96.5 (92.5-100.5) 58	92.6 (89.9-95.3) 117	90.5 (86.7-94.3) 77	95.6 (92.4-98.9) 84	89.6 (71.3-107.8) 7	89.8 (87-92.5) 114
Systolic blood pressure/mmHg	106.8 (105.7-107.9) 597	103.1 (96.6-109.5) 19	103.1 (96.6-109.5) 19	105.1 (101.6-108.6) 51	106.3 (104-108.5) 116	109.7 (106.8-112.5) 70	101.6 (98.3-104.8) 78	103.3 (90.6-116)6	106.6 (104.8-108.3) 99
Diastolic blood pressure/mmHg	67.9 (67.1-68.7) 596	69.2 (64.7-73.6) 19	64.45(60.2-68.8) 31	65.2 (62.4-68) 51	69.7 (68.2-71.2) 116	67.5 (65.3-69.7) 70	64.8 (62.4-67.2) 77	66 (57.6-74.4) 6	65.2 (63.8-66.7) 99
Respiratory, rate/min	23.1 (22.6-23.4) 617	23.3 (21.8-24.7) 16	24.3 (22.6-26) 29	22.5 (21.9-23) 58	23.9 (23.2-24.5) 103	23.9 (22.8-25.1) 75	23.9 (22.9-24.9) 73	21.1 (20.2-22.1) 7	22 (21.7-22.3) 111
GCS<15	17/624 (2.7)	0/15 (0)	1/29 (3.5)	2/56 (3.6)	2/94 (2.1)	22/72 (30.6)	2/72 (2.8)	0/7 (0)	0/113 (0)
Pale	135/652 (20.7)	0/18 (0)	10/29 (34.5)	12/59 (20.3)	8/112 (7.1)	11/78 (14.1)	23/83 (27.7)	1/7 (14.3)	13/114 (11.4)
Jaundice	34/650 (5.2)	3/19 (15.8)	3/30 (10)	4/59 (6.8)	4/112 (3.6)	2/78 (2.6)	7/82 (8.5)	0/7 (0)	4/114 (3.5)
Injected pharynx	82/645 (12.7)	1/18 (5.6)	2/29 (6.9)	8/58 (13.8)	14/111 (12.6)	4/76 (5.3)	6/83 (7.2)	1/7 (14.3)	13 (11.3)
Conjunctival suffusion	86/655 (13.1)	2/17 (11.8)	5/30 (16.7)	4/58 (6.9)	22/116 (19)	5/76 (6.6)	9/81 (11.1)	0/7 (0)	18 (15.7)
Rash	51/10879 (4.7)	0/20 (0)	1/40 (2.5)	2/106 (1.9)	9/143 (6.3)	6/106 (5.7)	10/113 (8.9)	1 (10)	3/115 (2.6)
Eschar	8/614 (1.3)	0/18 (0)	0/30 (0)	2/58 (3.5)	2/109 (1.8)	2/78 (2.6)	3/82 (3.7)	0/7 (0)	0/114 (0)
Hepatomegaly	28/608 (4.6)	1/16 (6.3)	5/29 (17.2)	3/52 (5.8)	2/110 (1.8)	2/67 (3)	10/79 (12.7)	0/7 (0)	1/107 (0.9)
Splenomegaly	17/606 (2.8)	0/16 (0)	0/28 (0)	4/53 (7.6)	2/110 (1.8)	1/67 (1.5)	8/79 (10.1)	0/7 (0)	1/107 (0.9)

Abnormal chest sounds	35/656 (5.3)	0/18 (0)	3/30 (10)	1/58 (1.7)	0/115 (0)	9/78 (11.5)	4/84 (4.8)	0/7 (0)	1 (0.9)
Lymphadenopathy	142/658 (21.6)	1/18 (5.6)	9/30 (30)	15/59 (25.4)	8/113 (7.1)	19/79 (24.1)	21/85 (24.7)	3/7 (42.9)	10/114 (8.8)
Seizure	17/661 (2.8)	1/19 (5.3)	0/30 (0)	1/59 (1.7)	2/118 (1.7)	19/80 (23.8)	1/84 (1.2)	0/7 (0)	0/113 (0)
Confusion	19/659 (2.9)	1/19 (5.3)	1/19 (5.3)	3/59 (5.1)	3/118 (1.5)	22/80 (27.5)	3/84 (3.6)	1/7 (14.3)	0/113 (0)
Neck stiffness	14/630 (2.2)	1/17 (5.9)	1/29 (3.5)	1/56 (1.8)	3/107 (2.8)	16/80 (20)	0/79 (0)	1/7 (14.3)	0/113 (0)
**Investigations**									
Haematocrit, %	40.2 (39.2-41.1) 316	41.4 (38.2-44.5) 12	39.6 (35.3-44) 15	39 (35.9-42.2) 35	40.7 (38.7-42.7) 52	41.5 (39.2-43.8) 40	36.6 (34.1-39.1) 44	42.2 (15.6-68.9) 3	44.3 (43.1-45.5) 87
WBC, x 109/L	10.31 (9.69-10.93) 319	5.86 (4.57-7.13) 11	8.58 (7.21-9.94) 15	10.2 (9.18-11.1) 35	6.55 (5.24-8.87) 52	11.03 (9.18-12.88)41	9.55 (8.39-10.7) 45	8.70 (3.66-13.74) 3	8.18 (6.49-9.88) 87
Neutrophils %	68.2 (66.5-69.9) 292	64.9 (58.5-71.3) 9	69.5 (63.2-75.9) 14	77.6 (72.1-83.1) 33	63 (55.6-70.5) 30	72.8 (68.2-77.4) 39	67.1 (63.3-70.8) 33	80 (80-80) 2	67.9 (65.1-70.6) 88
Lymphocytes, %	24.5 (22.2-26.7) 129	25.6 (16.9-34.4) 8	25.9 (20.6-31.3) 10	22.4 (17.3-27.6) 16	19.6 (14-25.2) 30	20.8 (16.9-24.7) 15	30 (26.8-32.2) 30	16.5 (5.9-27.1) 3	-
Platelets, x 109/L	243 (232-255) 243	174 (105-244) 10	226 (151-303) 9	194 (166-222) 22	216 (180-252) 41	227 (194-260) 28	230 (169-250) 34	280 1	209 (194-225) 82
CRP,/mg/L +	26.6 (1-846.9) 765	129.9 (12.8-318.9) 13	101 (1-608.6) 32	104.8 (1-558) 73	6.3 (1-238.7) 81	28.2 (1-381) 62	71.4 (2.2-349) 78	37.4 (2.7-208.3) 9	-

CRP >5mg/L	607/765 (79.4)	13/13 (100)	30/32 (93.8)	70/73 (95.9)	49/81 (60.5)	53/62 (85.5)	76/78 (97.4)	7/9 (77.8)	-

Supplementary Material-15. Comparison of fever aetiology between children and adults at Luang Namtha and Salavan. For patients with only evidence for one pathogen using conservative diagnoses. Influenza diagnosis was only conducted on samples collected from LNT for 6 months.

**Aetiology**	**Children 5 to ≤ 15 years (n = 540)**	**Adults > 15 years (n = 850)**	**P value**
**Luang Namtha**			
With diagnosis	193/540 (36%)	280/850 (33%)	0.283
Scrub typhus	31/516 (6%)	55/821 (6.7%)	0.616
JEV	33/535 (6.2%)	57/848 (6.7%)	0.684
Leptospirosis	54/539 (10%)	39/850 (4.6%)	<0.001
Bacteraemia	12/522 (2.3%)	18/816 (2.2%)	0.911
Dengue	6/535 (1.1%)	35/847 (4.1%)	0.001
Murine typhus	3/508 (0.6%)	3/812 (0.4%)	0.68
Multiple pathogens	34/540 (6.3%)	45/850 (5.3%)	0.432
Undetermined *Rickettsia* spp.	5/508 (1%)	3/812 (0.4%)	0.273

Malaria	0/540 (0)	4/850 (0.5%)	0.16
Influenza	49/127 (38.6%)	66/231 (28.6%)	0.052
**Salavan**			
With diagnosis	69/183 (37.7%)	156/365 (42.7%)	0.259
Scrub typhus	7/176 (4%)	29/358 (8.1%)	0.07
JEV	6/179 (3.4%)	16/362 (4.4%)	0.55
Leptospirosis	8/180 (4.4%)	8/365 (2.2%)	0.14
Bacteraemia	3/171 (1.8%)	10/321 (3.1%)	0.55
Dengue	38/182 (20.9%)	77/363 (21.2%)	0.93
Murine typhus	1/174 (0.6)	3/355 (0.9%)	1.00
Multiple pathogens	8/183 (4.4%)	14/365 (3.7%)	0.698
Undetermined *Rickettsia* spp.	0/174 (0%)	1/365 (0.3%)	1.00
Malaria	6/183 (3%)	12/365 (3%)	0.99

Supplementary Material-16. Univariable analysis and multivariable logistic regression analysis of predictors associated with detected pathogens. For patients with only evidence for one pathogen using conservative diagnoses. Univariate analysis only shown if P value ≤0.05 plus some clinically important variables+. Influenza diagnosis was only conducted on samples collected from LNT for 6 months.

**Variable**	**Sub category**	**Univariable**		**Multivariable**	
		**OR (95% CI)**	**P value**	**OR (95% CI)**	**P value**
**Influenza**					
Age	16-49 years	Reference		-	
	≤15 years	1.57 (0.9-2.4)	0.05		
Retro-orbital pain	No	Reference			
	Yes	1.9 (1-3.4)	0.02	2.1 (1.1-3.9)	0.018
Cough	No	Reference			
	Yes	4.0 (2.5-6.5)	<0.001	4.2 (2.6-6.7)	<0.001
Sore throat	No	Reference			
	Yes	1.8 (1.1-2.9)	0.02	-	-
Running nose	No	Reference			
	Yes	1.8 (1-3.2)	0.04	-	-
**Japanese Encephalitis**					
Province	SV	Reference			
	LNT	1.6 (1.0-2.6)	0.04	-	

Arthralgia	No	Reference			
	Yes	0.6 (0.4-0.9)	0.03	-	
GCS	15	Reference			
	<15	21.1 (10.9-40.6)	<0.001	4 (1.1-13.9)	0.03
Abnormal chest	No	Reference			
	Yes	3.1 (1.5-6.7)	0.003	-	
Seizure	No	Reference			
	Yes	16.9 (8.6-33.1)	<0.001	-	
Confusion	No	Reference			
	Yes	14.3 (7.8-26.3)	<0.001	-	
Neck stiffness	No	Reference			
	Yes	15.1 (8.2-27.8)	<0.001	-	
Drowsiness	No	Reference			
	Yes	14.3 (7.8-26.3)	<0.001	-	
AES	No	Reference			
	Yes	16 (9.2-27.9)	<0.001	5.6 (1.8-17.5)	0.003
**Dengue**					
Province	LNT	Reference			
	SV	8.7 (6-12.7)	<0.001	9.9 (4.8-20.2)	<0.001

Age	6-49	Reference			
	≤15	0.6 (0.4-0.9)	0.01	-	
Arthlagia	No	Reference			
	Yes	2.3 (1.5-3.6)	<0.001	-	
Back pain	No	Reference			
	Yes	0.5 (0.3-0.7)	<0.001	-	
Abdominal pain	No	Reference			
	Yes	0.3 (0.1-0.6)	0.001	-	
Cough	No	Reference			
	Yes	0.3 (0.2-0.5)	<0.001	-	
Pale	No	Reference			
	Yes	0.3 (0.1-0.6)	0.002	-	
Lymphadenopathy	No	Reference			
	Yes	0.3 (0.1-0.6)	0.001	-	
Tourniquet test positive	No	Reference			
	Yes	7.6 (3.3-17.3)	<0.001	-	
Platelets	>100,000/mm^3	Reference			
	≤100,000/ mm^3	2.7 (1.1-7.1)	0.03	4 (1.4-11.5)	0.01

Bacteraemia					
Arthralgia	No	Reference			
	Yes	0.3 (0.2-0.7)	0.005	0.4 (0.2-0.9)	0.03
Vomiting	No	Reference			
	Yes	1.9 (1-3.5)	0.04	-	
Diarrhoea	No	Reference			
	Yes	3.1 (1.6-6)	0.001	-	
Abdominal pain	No	Reference			
	Yes	2.8 (1.3-5.9)	0.006	2.4 (1.1-5.2)	0.03
Dysuria	No	Reference			
	Yes	3.1 (1.1-9.1)	0.03	-	
Sore throat	No	Reference			
	Yes	0.3 (0.1-0.9)	0.03	-	
Pale	No	Reference			
	Yes	2.4 (1.1-5.2)	0.03	-	
Hepatomegaly	No	Reference			
	Yes	4.7 (1.7-12.8)	0.003	3.6 (1.2-10.4)	0.02
**Leptospirosis**					

Province	SV	Reference			
	LNT	2.4 (1.4-4.1)	0.002	1.9 (1-3.5)	0.04
Age	16-49	Reference			
	≤15	2.3 (1.6-3.5)	<0.001	2.2 (1.3-3.6)	0.002
Gender	Female	Reference			
	Male	1.9 (1.3-3)	0.002	-	
Vomiting	No	Reference			
	Yes	2.7 (1.9-4.1)	<0.001	2.2 (1.3-3.6)	0.003
Cough	No	Reference			
	Yes	0.4 (0.2-0.7)	0.001	0.2 (0.1-0.5)	<0.001
CRP	≤5mg/L	Reference			
	>5mg/L	5.5 (1.7-17.6)	0.004	5.3 (1.6-17.2)	0.005
**Scrub typhus**					
Gender	Male	Reference			
	Female	1.5 (1.0-2.1)	0.04	1.8 (1.1-2.8)	0.01
Constipation	No	Reference			
	Yes	2.7 (1.3-5.4)	0.007	-	
Pale	No	Reference			
	Yes	1.8 (1.1-2.9)	0.02	-	

Rash	No	Reference			
	Yes	2.2 (1.1-4.3)	0.03	-	
Hepatomegaly	No	Reference			
	Yes	3.4 (1.6-7)	0.001	3.4 (1.6-7.2)	0.001
Splenomegaly	No	Reference			
	Yes	4.4 (1.9-10.1)	<0.001	-	
CRP	≤5mg/L	Reference			
	>5mg/L	9.1 (2.2-37.5)	0.002	-	

Supplementary Material-17. Clinical presentation and diagnosis of patients known to have died (n = 6)

Code	Age (yrs)	Sex	Days between admission and death	Admission impression	Signs and symptoms on admission	Diagnosis
SV-380	13	M	Admitted 25-Sep-10 & died the same day	Septic shock	Illness for 60 days with fistulae on the right thigh > one month. Admitted with fever ~ 5 days, headache, myalgia, dyspnoea, agitation, drowsiness. Temp: 38.0^{0}C, pulse: 196, BP: 80/50, RR: 40. GCS: 15/15	Melioidosis confirmed by haemoculture
LNT-947	14	M	Admitted 12-Jun-09 & died on 15-Jun-09	Meningo-encephalitis	Illness for 5 days, fever~2 days, chill, headache, myalgia, nausea, vomiting, convulsion, neck stiffness, agitation, drowsiness. Temp: 39.3^{0}C, pulse: 117, BP: 126/62, RR: 24, GCS~10/15.	No diagnosis made
LNT-1366	17	M	Admitted 9-Apr-10 & died the same day	Myositis, bacteraemia, meningitis	Seven days of illness, fever~5 days, headache, back pain, myalgia, nausea, vomiting, agitation, drowsiness, abdominal pain, abscess~3 cm on left thigh. Temp: 39.0^{0}C, pulse: 120, BP: 88/46, RR: 24, GCS: 15/15. Left inguinal lymph node, hepatomegaly~2 cm, abdominal tenderness, neck stiffness.	Bacteraemia (*S. aureus* in blood culture)
LNT-1511	10	M	Admitted 10-Jul-10 & died on 15-Jul-10	Meningo-encephalitis	Illness ~ 8 days, fever ~ 8 days, headache, back pain, myalgia, nausea, vomiting, cough, dyspnoea, convulsion, neck stiffness, agitation, drowsiness. Temp: 38.5^{0}C, pulse: 125, BP: 112/70, RR: 50. Lungs: bilateral crepitations. GCS~5/15.	JE as confirmed by ELISA
LNT-1332	18	F	Admitted 28-Jan-10 & died on 1-Feb-10	Encephalitis	Five days of illness, fever~4 days, chill, headache, nausea, neck stiffness, confusion, drowsiness, abdominal pain. Temp: 38.0^{0}C, pulse: 67, RR: 26, BP: N/A, GCS: 13/15.	Dengue as confirmed by NS1 positive

Supplementary Material-18. Peripheral blood white cell counts (WCC) and C-reactive protein (CRP)

Median WCCs, when available, were significantly higher (P<0.001) for those patients with a bacterial pathogen and for those with an aetiology that would be expected to respond to doxycycline than those not expected to respond (Supplementary Material-20). With a WCC cut off of $\geq 11 \times 10^9$/L, more patients had raised WCC in the bacterial than viral group and among the patients expected to respond to doxycycline, but the differences were not significant (P=0.27 & 0.068).

Although median CRP concentrations were lowest for patients with JEV, dengue and murine typhus, the percentage of patients with CRP >5 mg/L was >50% for all diagnostic groups. Median CRP and % CRP >5 mg/L were significantly higher for those with bacterial than viral diseases (P<0.001)(below, Fig. 7). The sensitivity, specificity, positive predictive value (PPV) and negative predictive value (NPV) of CRP≥5mg/L for bacterial infection versus confirmed viral infection were 94.4, 20.7, 19.5 and 94.8 %, respectively. Including all patients with and without diagnoses, the sensitivity, specificity, positive predictive value (PPV) and negative predictive value (NPV) of CRP>5mg/L for bacterial infection were 94.4, 20.7, 21.1 and 94.3%, respectively. The median CRP and % CRP >5 mg/L for those infected with pathogens likely to respond to doxycycline were also significantly higher than those infected with pathogens unlikely to respond (P<0.001)(below). The sensitivity, specificity, PPV and NPV of CRP≥5mg/L for an infection likely to respond to doxycycline were 94.5, 20.2, 16.2 and 95.8 %, respectively.

Relationship between peripheral blood white cell count (WCC) and serum CRP and bacterial *versus* viral aetiology and aetiology likely and not likely to respond to doxycycline. For patients with evidence for only one pathogen with conservative diagnoses.

	**Bacterial aetiology ***	**Viral aetiology****	**P value**
**Median (range) WCC 10^9/L**	10.0 (2.7-98.0)	6.6 (1.2-58.0)	<0.001
**WCC $\geq$11*10^9 /L**	24/99 (24%)	34/182 (19%)	0.271
	**Likely to respond to doxycycline #**	**Unlikely to respond to doxycycline ##**	
**Median (range) WCC**	10.0 (2.7-98.0)	6.7 (1.2-58.0)	<0.001
**WCC $\geq$11*10^9 /L**	23/84 (27%)	35/197 (18%)	0.068

	**Bacterial aetiology ***	**Viral aetiology****	**P value**
**Median (range) CRP mg/L**	77.9 (1-608.6)	9.2 (1-381)	<0.001
**CRP $\geq$5 mg/L**	187/198 (94%)	102/143 (71%)	<0.001
	**Likely to respond to doxycycline #**	**Unlikely to respond to doxycycline ##**	
**Median (range) CRP mg/L**	77.3 (1-558)	16.5 (1-608.6)	<0.001

**CRP ≥5 mg/L**	156/165 (95%)	133/176 (76%)	<0.001

* Bacterial aetiology = bacteraemia, leptospirosis, scrub typhus, murine typhus and undetermined typhus; ** Viral aetiology = dengue fever, Japanese encephalitis virus infection and influenza;

Likely to respond to doxycycline = leptospirosis, scrub typhus, murine typhus and undetermined typhus; ## Unlikely to respond to doxycycline = Japanese encephalitis virus infection, dengue fever, influenza and bacteraemia

Supplementary Material-19. Antibiotics used by patients at LNT Provincial Hospital by diagnosis – for patients with only evidence for one pathogen using conservative diagnoses. Some patients received >1 antibiotic. Red indicates that the antibiotic may have some efficacy

Antibiotics	Total	LNT - diagnosis						
		Bacteraemia	Typhus	Malaria	Leptospirosis	Influenza	Dengue	JEV
Any antibiotic therapy /patient with data * (%)	560/1,095 (51)	14/28 (50)	52/89 (58)	2/3 (67)	45/79 (57)	9/25 (36)	15/31 (48)	52/73 (71)
Ampicillin	3	1	0	0	0	1	0	0
Amoxicillin	294	7	23	1	25	6	7	20
Cephalothin	0	0	0	0	0	0	0	0
Penicillin	13	5	1	0	1	0	1	2
Ceftriaxone	87	2	8	1	8	1	1	20
Cloxacillin	4	0	0	0	0	0	0	0
Erythromycin	5	0	1	0	0	0	0	1
Doxycycline	116	4	16	0	7	1	2	7
Azithromycin	4	0	0	0	0	0	0	0
Ofloxacin	45	1	5	0	4	0	4	1
Lincomycin	1	0	0	0	0	0	0	0
Co-trimoxazole	5	0	1	0	0	0	0	0
Metronidazole	10	0	1	0	0	0	1	2
Gentamicin	45	2	4	1	0	0	1	10
Potentially appropriate therapy for all patients with data (%)	77/1,095 (7)	12/28 (43)	16/89 (18)	0/3 (0)	41/79 (52)	0/25 (0)	0/31 (0)	0/73 (0)

Supplementary Material - 20. Estimated pathogen specific expected therapeutic responses.
The actual efficacy will depend on dose, duration, the pathogen and the host. These are broad generalisations and should not be used to guide individual patient therapy. Please see references at table base that were used to make an author consensus

	Oral doxycycline	Oral ofloxacin	Oral azithromycin	Iv ceftriaxone	Artemether-lumefantrine
Malaria	minimal	0	minimal	0	~ 97% [a]
Dengue	0	0	0	0	0
JEV	0	0	0	0	0
Scrub typhus	~ 97% [b,c]	minimal [d]	90-100% [b,c] assumed 95%	0	0
Murine typhus	≤100% [b]	uncertain	uncertain	0	0
SFG *Rickettsia* spp.	Probably ≤100%	uncertain	uncertain	0	0
Leptospirosis	~ 97% [c]	uncertain [e]	~ 97% [c]	≤100%	0
Community-acquired bacteraemia[f,g]	minimal	~ ≤94%	Good [h] assumed to be 75%	≤ 100 % inadequate for *B. pseudomallei* [g]	0

Based on:

a Mayxay *et al.* (2010)[2] **; b** Unpublished clinical trial data from Mahosot Hospital; c Phimda *et al.* (2007)[28]; d Tantibhedhyangkul *et al.* (2010); e Griffith *et al.* (2006); f Phongmany *et al.* (2005); g See Tables 2 for pathogens, Phetsouvanh *et al.* (2006)3; h Parry *et al.* (2007); g Chaowagul *et al.* (1999)

Additional References

Blacksell SD, Khounsy S, Phetsouvanh R, Newton PN. A simple and inexpensive container for the transport of biological specimens in limited resource situations. *Trans R Soc Trop Med Hyg.* 2006; **100**: 1084-6.

Coleman RE, Sangkasuwan V, Suwanabun N, Eamsila C, Mungviriya S, Devine P, et al. Comparative evaluation of selected diagnostic assays for the detection of IgG and IgM antibody to *Orientia tsutsugamushi* in Thailand. *Am J Trop Med Hyg.* 2002; **67:** 497-503.

Jacobson JA, Hills SL, Winkler JL, Mammen M, Thaisomboonsuk B, Marfin AA, et al. Evaluation of three immunoglobulin M antibody capture enzyme-linked immunosorbent assays for diagnosis of Japanese encephalitis. *Am J Trop Med Hyg.* 2007; **77:** 164-8

Lanciotti RS, Calisher CH, Gubler DJ, Chang GJ, Vorndam AV. Rapid detection and typing of dengue viruses from clinical samples by using reverse transcriptase-polymerase chain reaction. J Clin Microbiol. 1992; 30: 545-51.

Levett PN. Leptospirosis. *Clin Microbiol Rev.* 2001; **14:** 296-326.

Ninove L, Nougairede A, Gazin C, Thirion L, Delogu I, Zandotti C, et al. RNA and DNA bacteriophages as molecular diagnosis controls in clinical virology: a comprehensive study of more than 45,000 routine PCR tests. PLoS One. 2011; 6: e16142.

Phongmany S, Phetsouvanh R, Sisouphone S, Darasavath C, Vongphachane P, Rattanavong O, et al. A randomized comparison of oral chloramphenicol versus ofloxacin in the treatment of uncomplicated typhoid fever in Laos. *Trans R Soc Trop Med Hyg.* 2005; **99**: 451-8.

Steenkeste N, Incardona S, Chy S, Duval L, Ekala MT, Lim P, et al. Towards high-throughput molecular detection of *Plasmodium*: new approaches and molecular markers. Malar J. 2009; 8: 86.

Trudnowski RJ, Rico RC. Specific gravity of blood and plasma at 4 and 37 degrees C. *Clin Chem.* 1974; **20**: 615-6.

Smythe LD, Wuthiekanun V, Chierakul W, Suputtamongkol Y, Tiengrim S, Dohnt MF, et al. The microscopic agglutination test (MAT) is an unreliable predictor of infecting Leptospira serovar in Thailand. *Am J Trop Med Hyg.* 2009; **81**: 695-7.

Tantibhedhyangkul W, Angelakis E, Tongyoo N, Newton PN, Moore CE, Phetsouvanh R, et al. Intrinsic fluoroquinolone resistance in *Orientia tsutsugamushi*. *Int J Antimicrob Agents.* 2010; **35**: 338-41.

Griffith ME, Hospenthal DR, Murray CK. Antimicrobial therapy of leptospirosis. *Curr Opin Infect Dis.* 2006; **19**: 533-7.

Parry CM, Ho VA, Phuong le T, Bay PV, Lanh MN, Tung le T, et al. Randomized controlled comparison of ofloxacin, azithromycin, and an ofloxacin-azithromycin combination for treatment of multidrug-resistant and nalidixic acid-resistant typhoid fever. *Antimicrob Agents Chemother*. 2007; **51**: 819-25.

Chaowagul W, Simpson AJ, Suputtamongkol Y, White NJ. Empirical cephalosporin treatment of melioidosis. *Clin Infect Dis*. 1999; **28**: 1328.

Supplementary Figure-1 Map of Luang Namtha (top) and Salavan (bottom) showing the location of homes of patients recruited.
H = provincial hospital

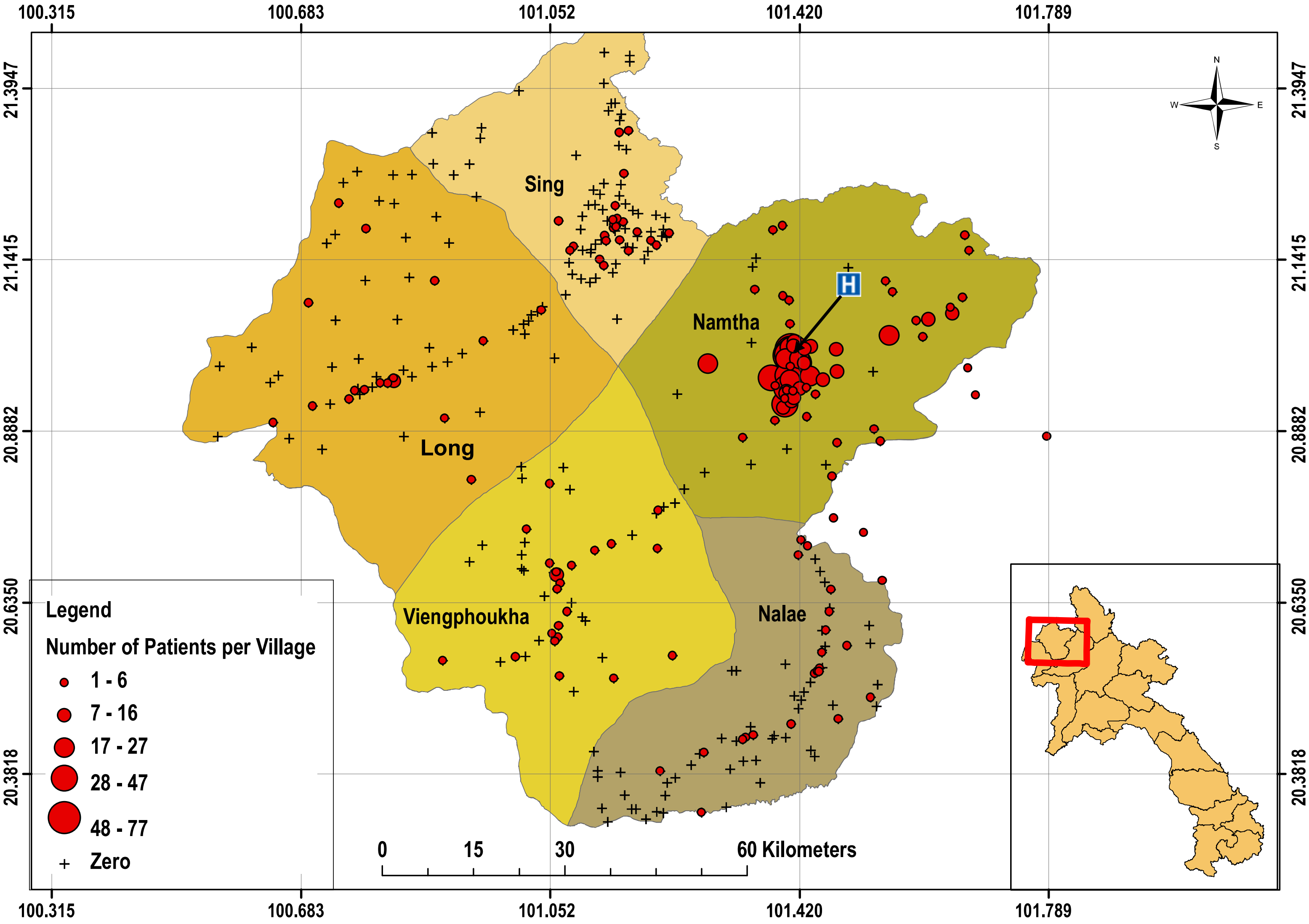

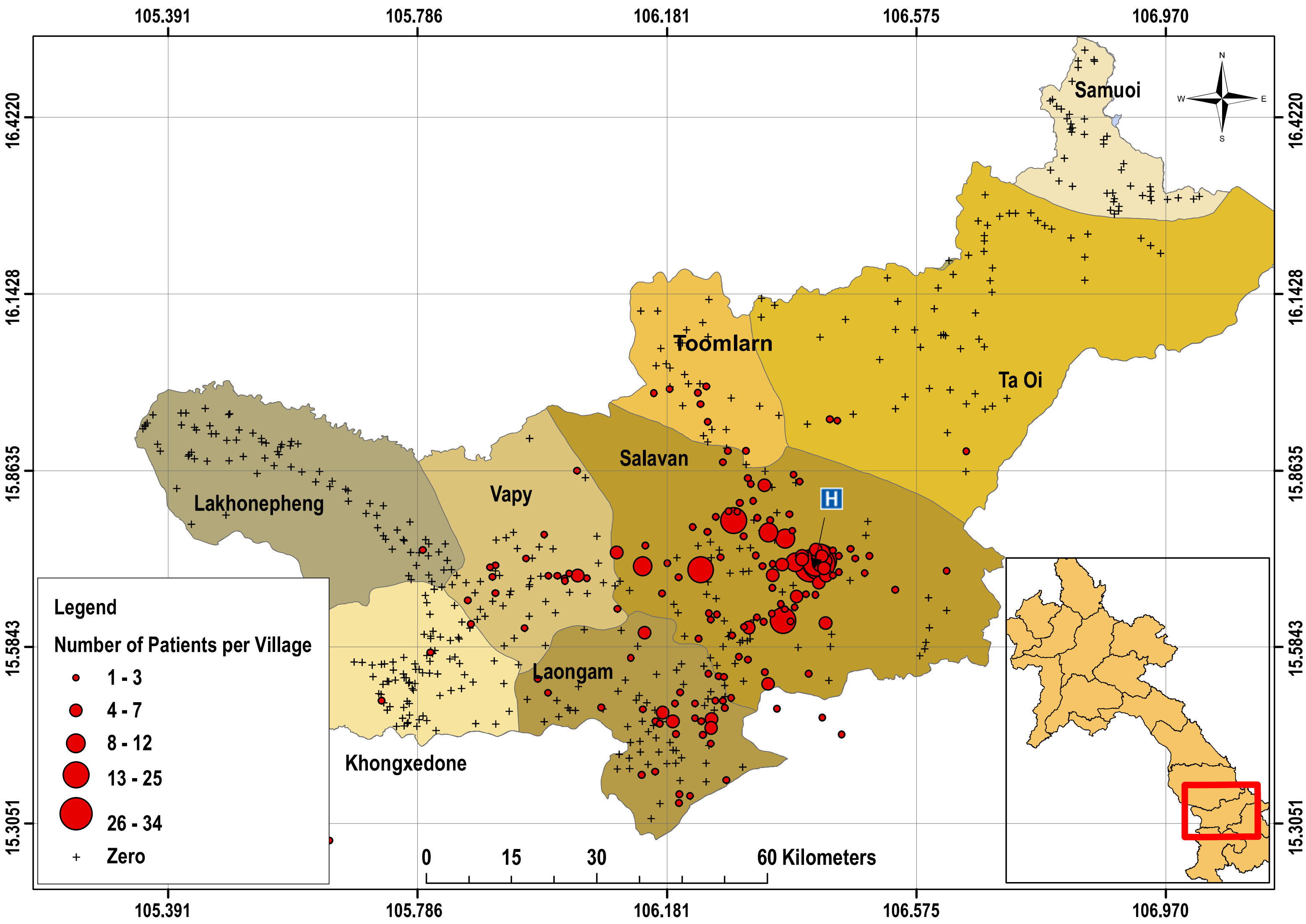
105.391
105.786
106.181
106.575
106.970
16.4220
16.1428
15.8635
15.5843
15.3051
Samuoi
Toomlarn
Ta Oi
Salavan
Vapy
Lakhonepheng
Laongam
Khongxedone
H
Legend
Number of Patients per Village
1 - 3
4 - 7
8 - 12
13 - 25
26 - 34
Zero
0
15
30
60 Kilometers

Supplementary Figure-2. Frequency distribution of the estimated distance of patients' homes from the provincial hospitals

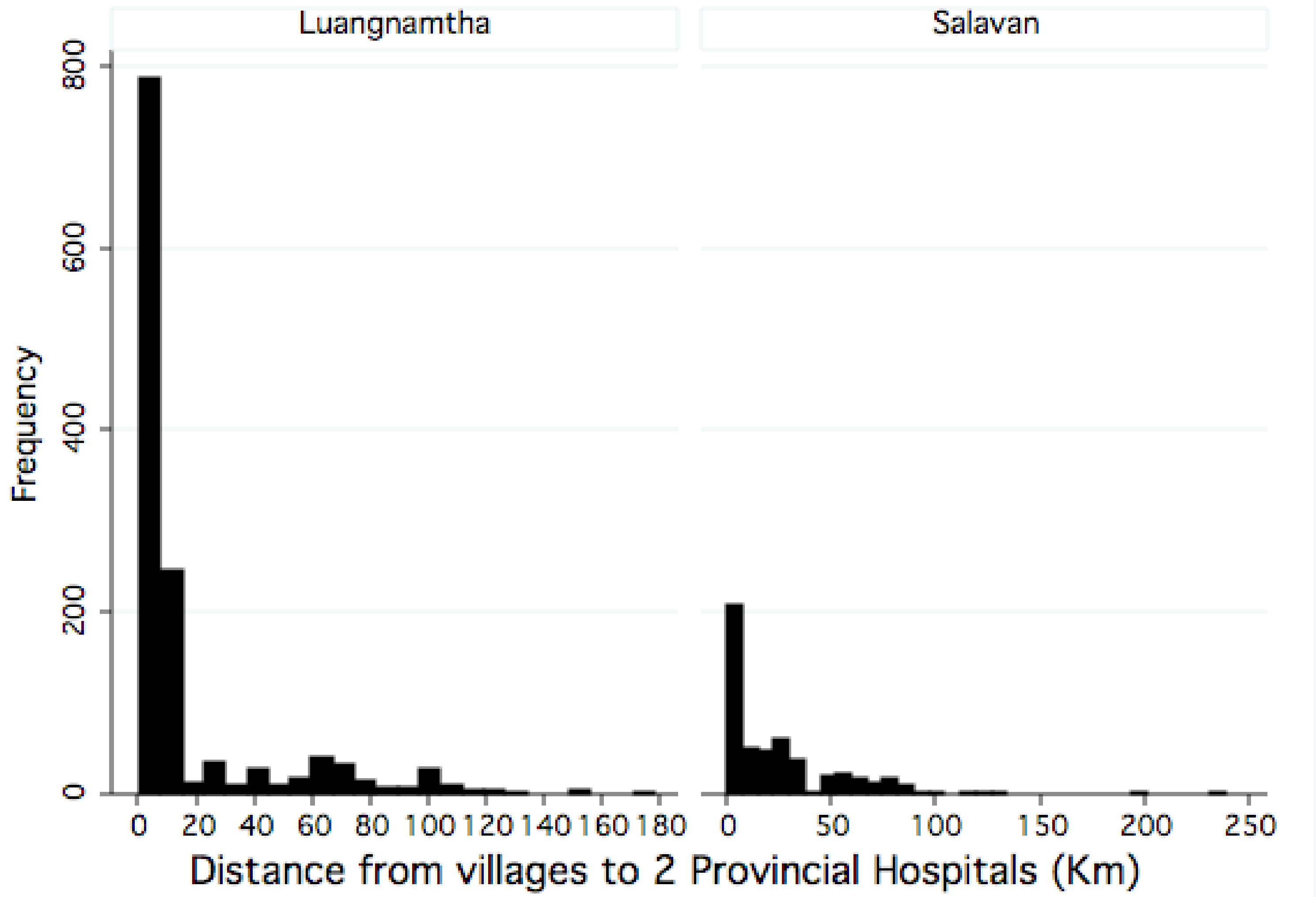

Supplementary Figure-3. Age distribution of the patients enrolled in the study. The red vertical line shows the age of 15 years

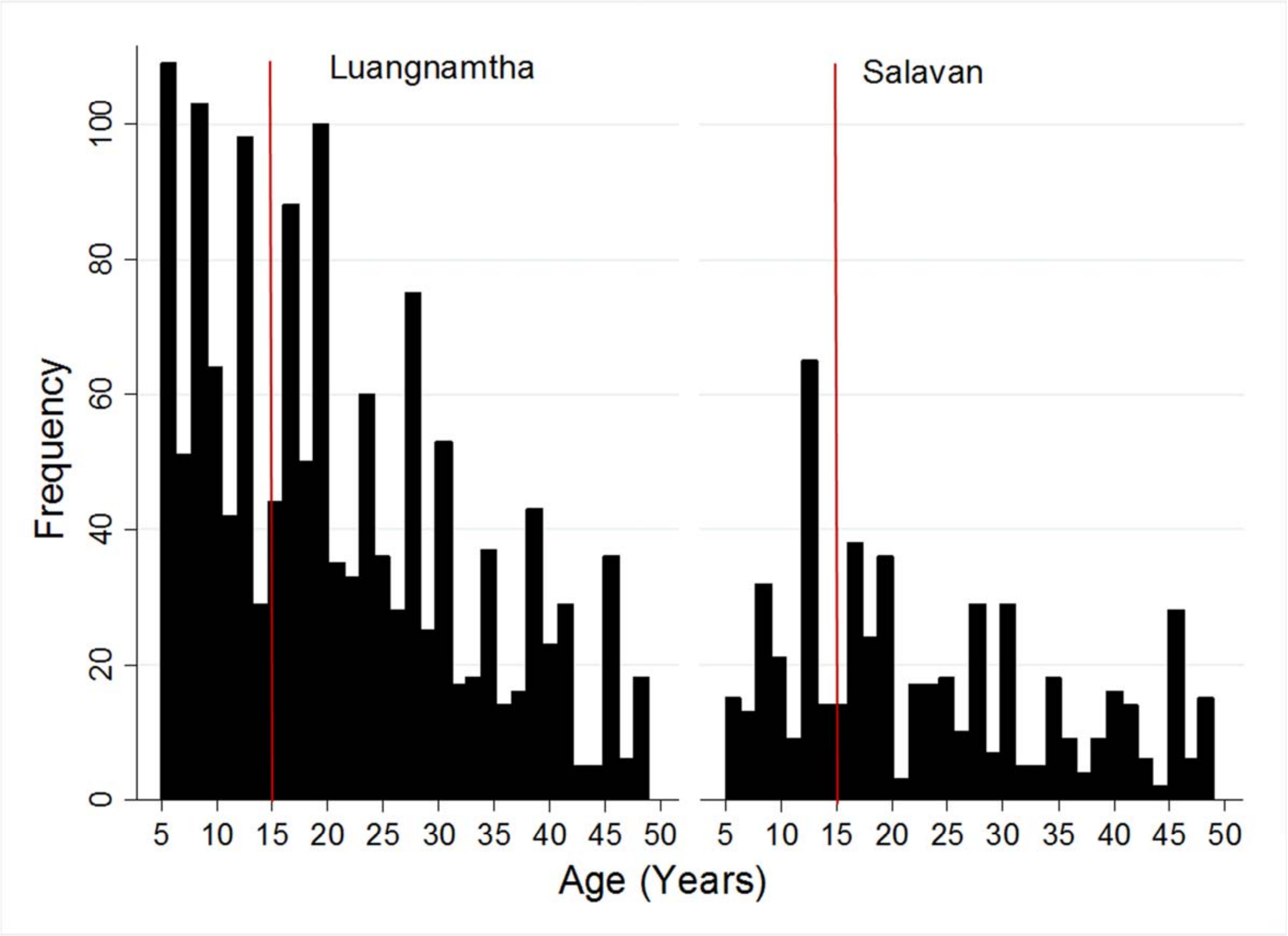

Supplementary Figure 4A. Pie chart of the diagnoses for the patients from LNT using only culture, antigen and nucleic acid detection assays (conservative definition) (n = 546). Influenza diagnosis was only conducted on samples collected from LNT for 6 months.

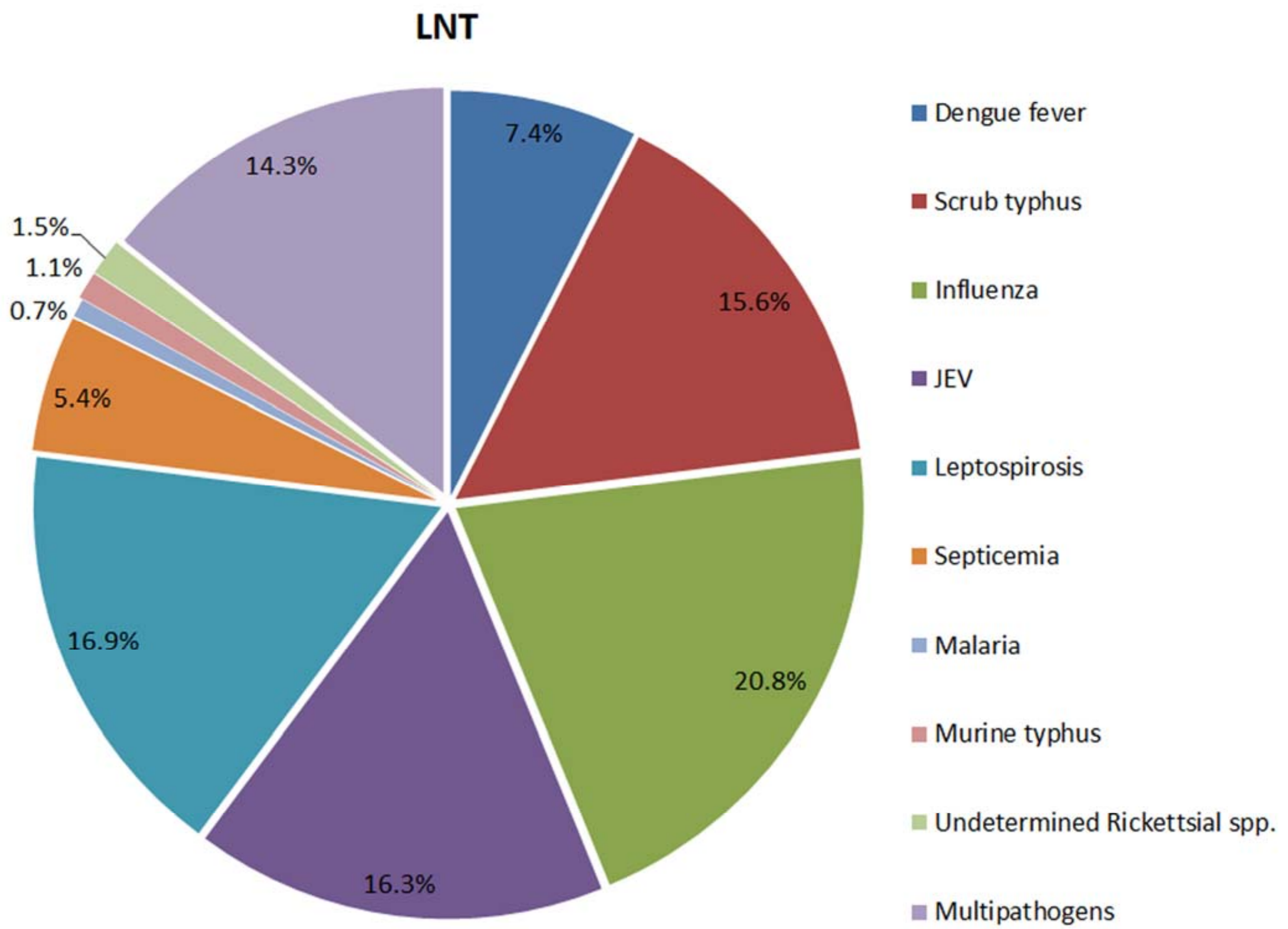

Supplementary Figure 4B. Pie chart of the diagnoses for the patients from SV using only culture, antigen and nucleic acid detection assays (conservative definition) (n = 247).

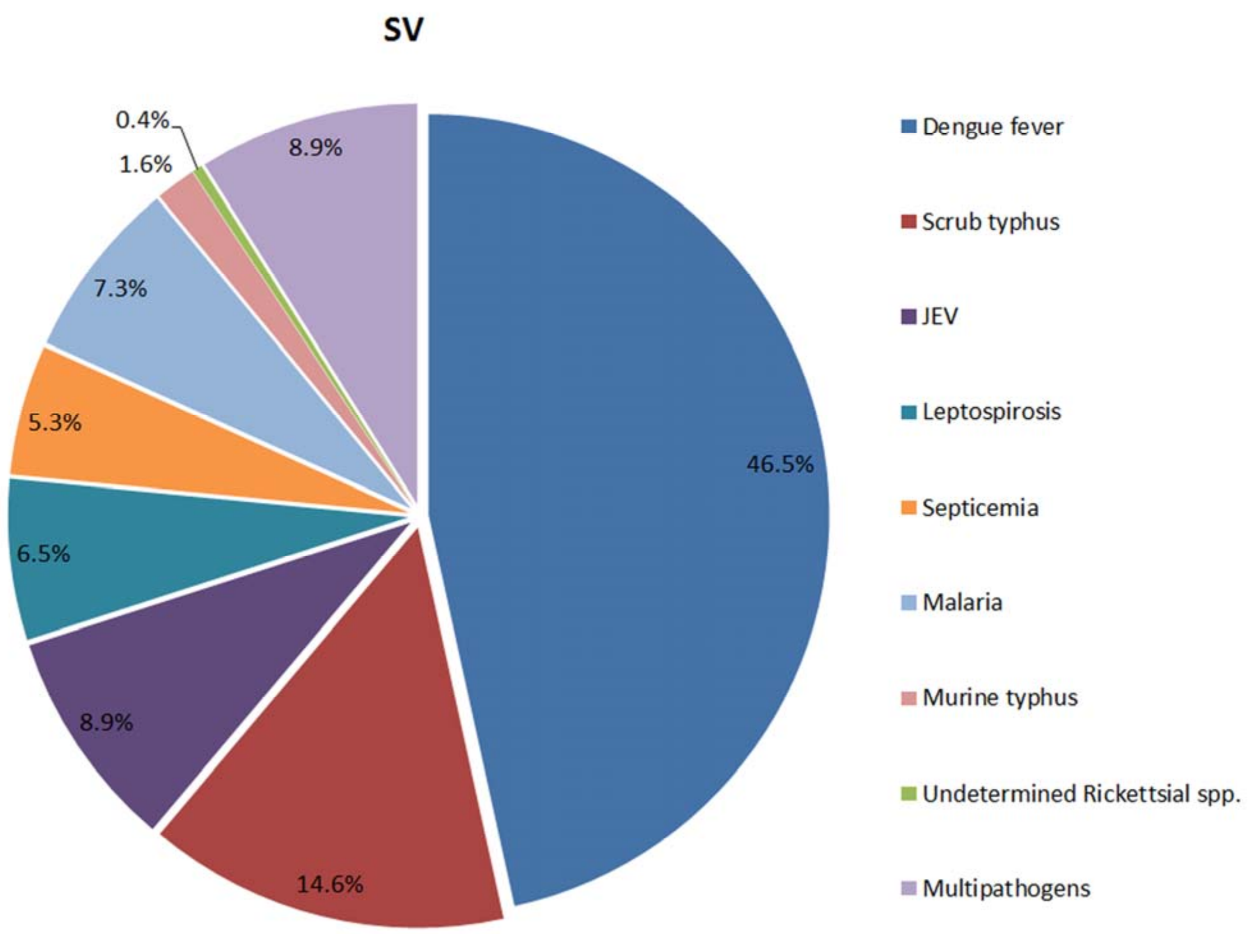

Supplementary Figure-5. Distribution of IgM (top) and IgG (bottom) against *O. tsutsugamushi* (blue) and *R. typhi* (red) (IFA)

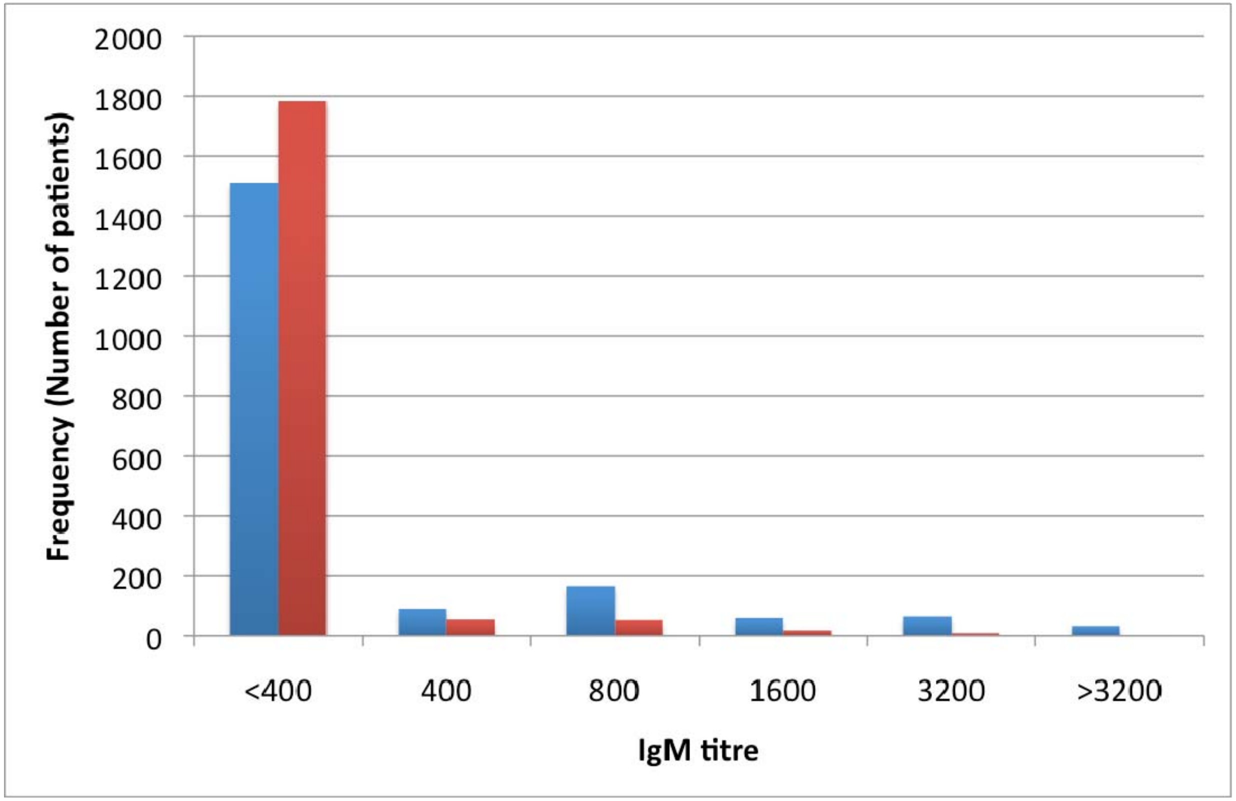

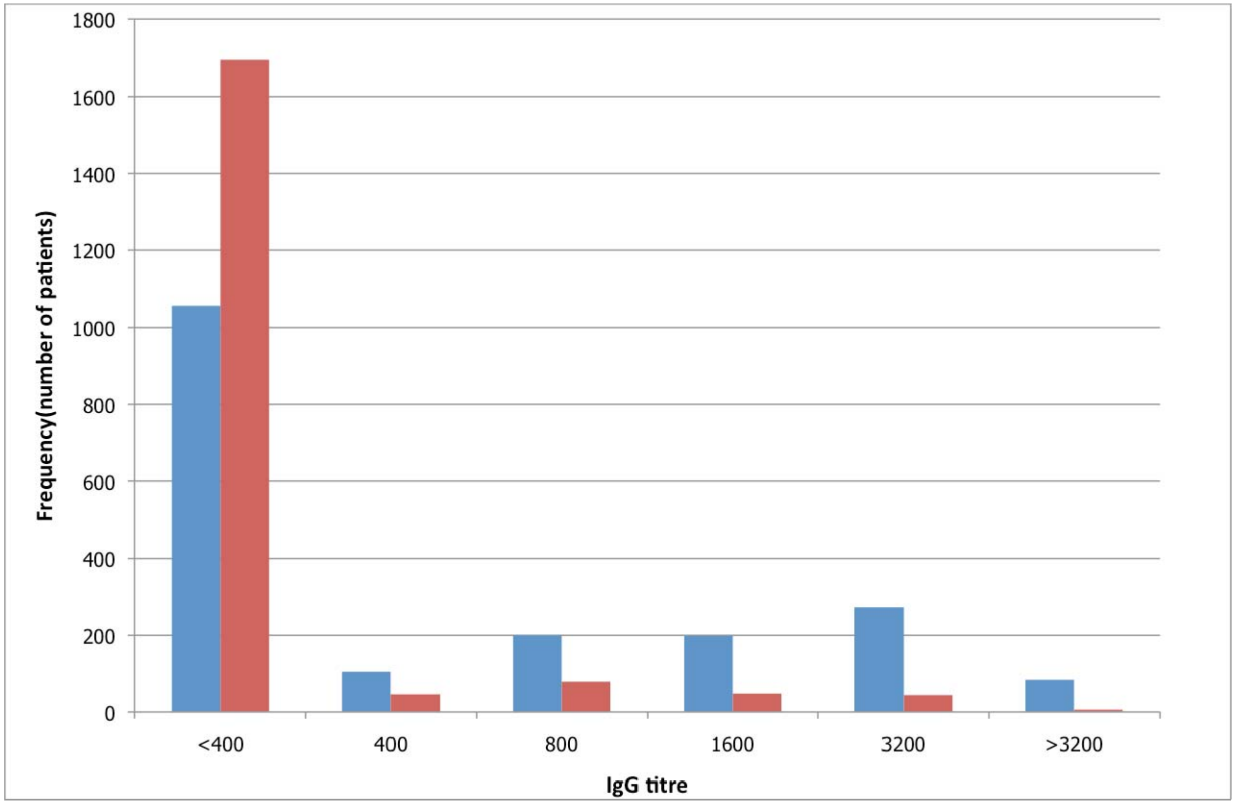

1800
1600
1400
1200
1000
800
600
400
200
0
Frequency(number of patients)
<400
400
800
1600
3200
>3200
IgG titre

Supplementary Figure 6. Monthly variation in number patients recruited throughout the study of 28 months at SV and 32 months and LNT

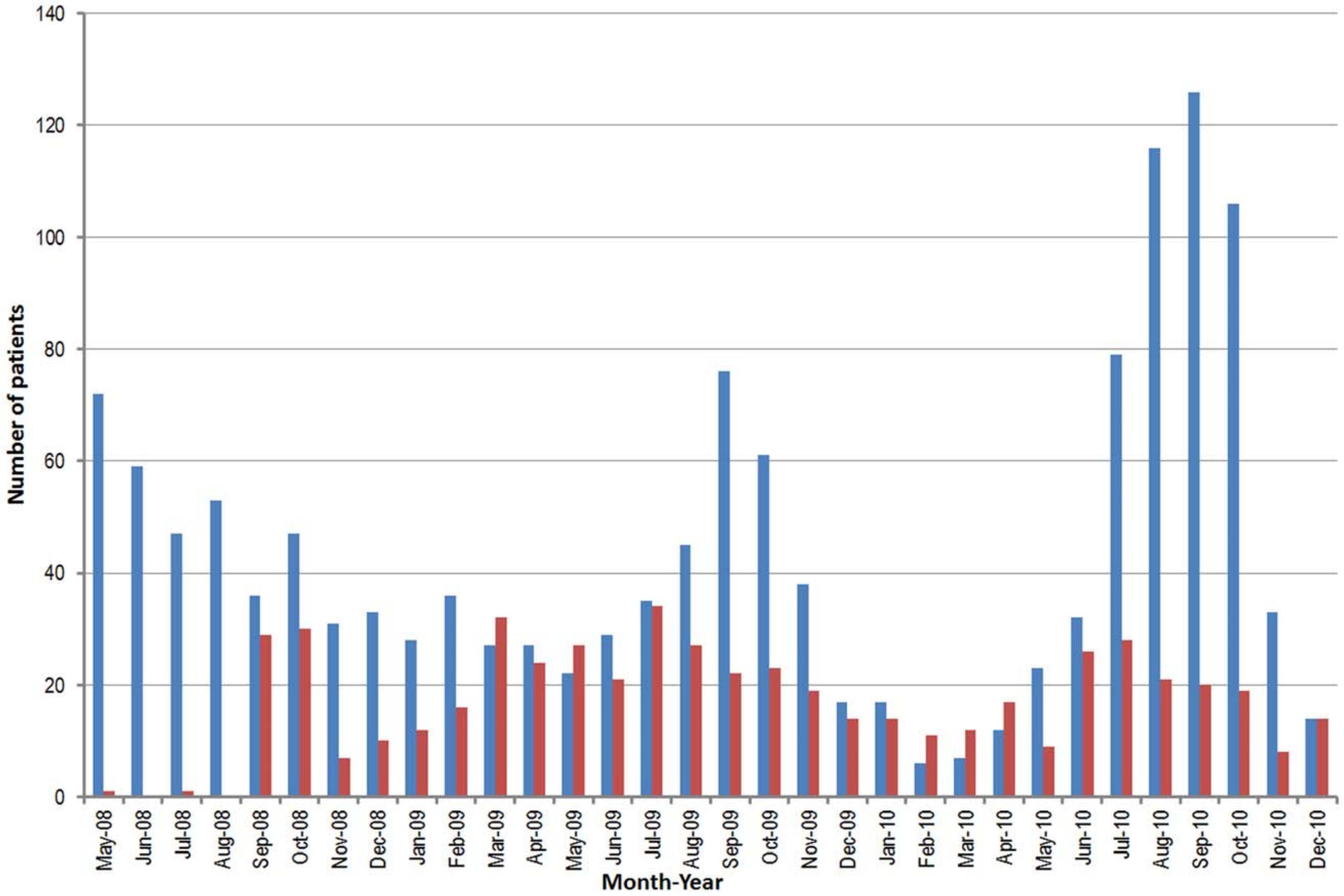

Supplementary Figure 7. Box and whisker plot for CRP concentration for those without (median 27.1 (1-846.9) mg/L) and with doxycycline responsive illness (DRI) (median 73.3 (1-558) mg/L) (DRI = scrub typhus, murine typhus, SFG and leptospirosis). For only patients with monopathogens, using only culture, antigen and nucleic acid detection assays plus anti-JEV IgM (conservative definition)

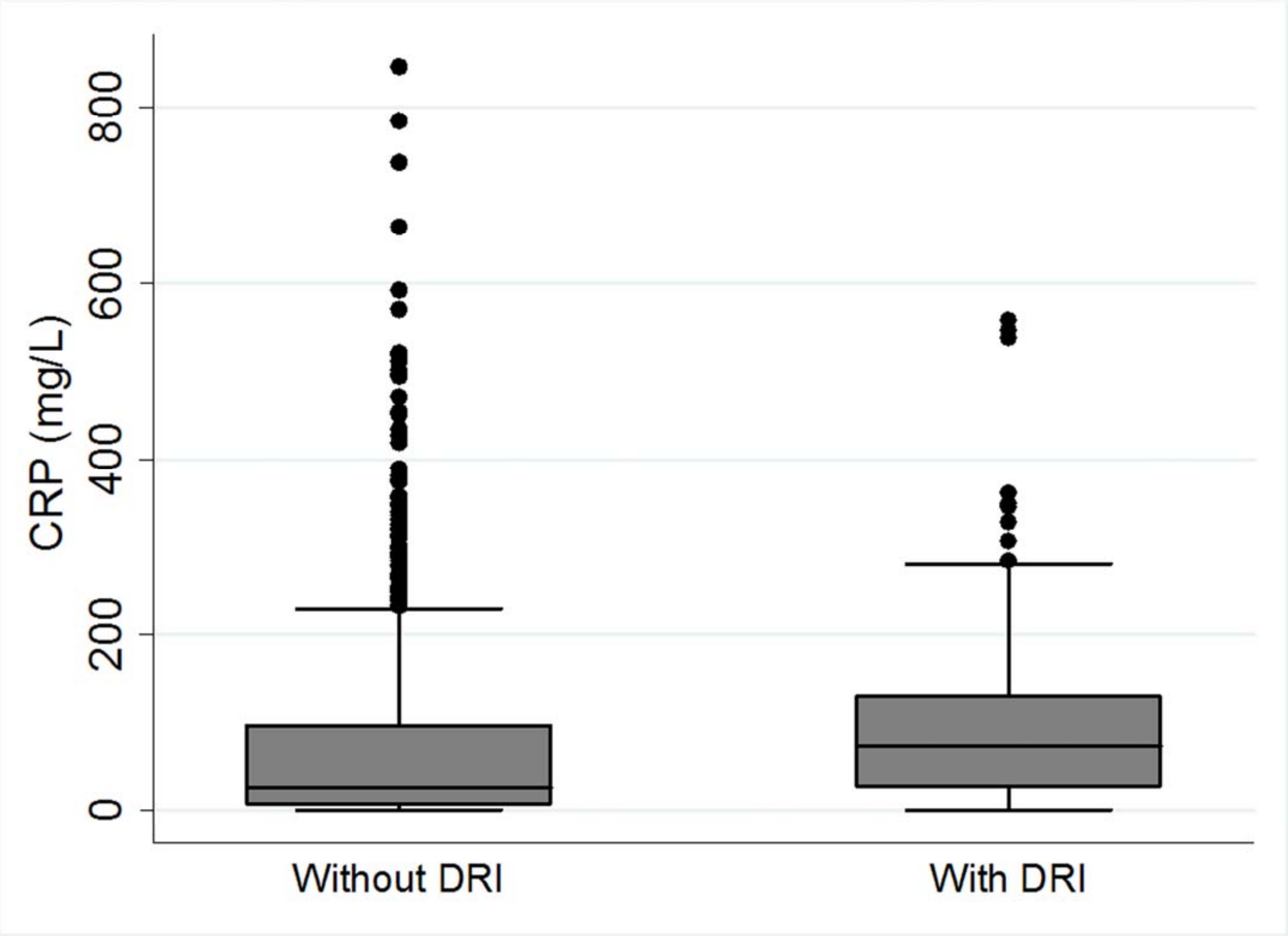

Supplement: Supplementary appendix [file mmc1.pdf]
